# DNA hypomethylation silences antitumor immune genes in early prostate cancer and CTCs

**DOI:** 10.1016/j.cell.2023.05.028

**Published:** 2023-06-15

**Authors:** Hongshan Guo, Joanna A. Vuille, Ben S. Wittner, Emily M. Lachtara, Yu Hou, Maoxuan Lin, Ting Zhao, Ayush T. Raman, Hunter C. Russell, Brittany A. Reeves, Haley M. Pleskow, Chin-Lee Wu, Andreas Gnirke, Alexander Meissner, Jason A. Efstathiou, Richard J. Lee, Mehmet Toner, Martin J. Aryee, Michael S. Lawrence, David T. Miyamoto, Shyamala Maheswaran, Daniel A. Haber

**Affiliations:** 1. Massachusetts General Hospital Cancer Center, Harvard Medical School, Charlestown, MA 02129, USA.; 2. Howard Hughes Medical Institute, Chevy Chase, MD 20815, USA.; 3. Evergrande Center for Immunologic Diseases, Brigham and Women’s Hospital, Harvard Medical School, Boston, MA 02115, USA.; 4. Broad Institute of MIT and Harvard, Cambridge, MA 02142, USA.; 5. Department of Pathology, Massachusetts General Hospital, Harvard Medical School, Boston, MA 02114, USA.; 6. Department of Radiation Oncology, Massachusetts General Hospital, Harvard Medical School, Charlestown, MA 02129, USA.; 7. Department of Genome Regulation, Max Planck Institute for Molecular Genetics, Berlin 14195, Germany.; 8. Department of Medicine, Massachusetts General Hospital, Harvard Medical School, Boston, MA 02114, USA.; 9. Department of Surgery, Massachusetts General Hospital, Harvard Medical School, Boston, MA 02114, USA.; 10. Center for Engineering in Medicine and Shriners Hospital for Children, Harvard Medical School, Boston, MA 02114, USA.; 11. Present address: Bone Marrow Transplantation Center, First Affiliated Hospital, Zhejiang University School of Medicine and Liangzhu Laboratory, Zhejiang University Medical Center, Hangzhou, 310012, China.; 12. Present address: Department of Data Science, Dana-Farber Cancer Institute, Harvard Medical School, Boston, MA 02114, USA.; 13. These authors contributed equally.; 14. Lead contact.

**Keywords:** DNA hypomethylation, prostate cancer, circulating tumor cells, immune surveillance, single-cell sequencing

## Abstract

Cancer is characterized by hypomethylation-associated silencing of large chromatin domains, whose contribution to tumorigenesis is uncertain. Through high-resolution genome-wide single-cell DNA methylation sequencing, we identify 40 core domains that are uniformly hypomethylated from earliest detectable stages of prostate malignancy through metastatic Circulating Tumor Cells (CTCs). Nested among these repressive domains are smaller loci with preserved methylation that escape silencing and are enriched for cell proliferation genes. Transcriptionally silenced genes within the core hypomethylated domains are enriched for immune-related genes; prominent among these is a single gene cluster harboring all five *CD1* genes that present lipid antigens to NKT cells, and four *IFI16*-related interferon-inducible genes implicated in innate immunity. Re-expression of *CD1* or *IFI16* murine orthologs in immunocompetent mice abrogates tumorigenesis, accompanied by activation of anti-tumor immunity. Thus, early epigenetic changes may shape tumorigenesis, targeting co-located genes within defined chromosomal loci. Hypomethylation domains are detectable in blood specimens enriched for CTCs.

## In Brief

Analysis of circulating tumor cluster cells reveals how DNA hypomethylation during early prostate tumorigenesis silences immune surveillance genes, while sparing proliferation-associated genes.

## Graphical Abstract

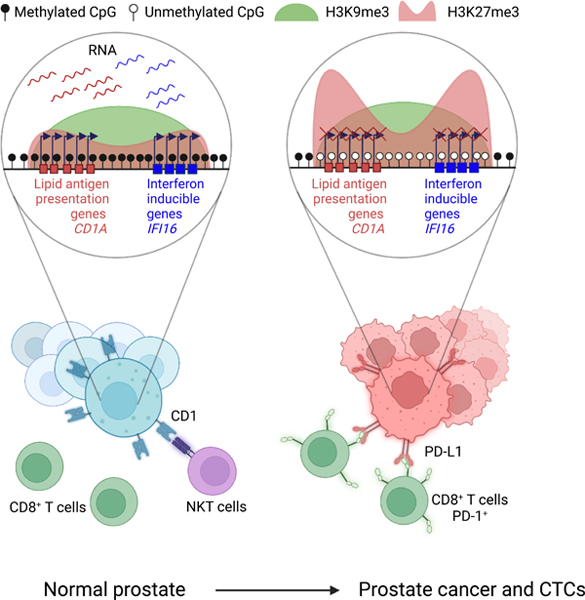

## Introduction

Cancer is characterized by two primary changes at the level of DNA methylation^1–4^. Focal hypermethylation of CpG islands, often located within gene regulatory regions, results in gene silencing, a well-established mechanism for inactivation of tumor suppressor genes^5–7^. In addition, long-range hypomethylated regions, Partially Methylated Domains (PMDs), coincide with nuclear Lamina-Associated Domains (LADs) and Large Organized Chromatin lysine (K) (LOCK) domains^8–10^. These chromosomal loci are large (>100 kb), gene-poor, correlated with late-replicating DNA, and topologically associated with nuclear lamina. Repetitive sequences and retro-elements residing within PMDs may be de-repressed in cancer, but the rare protein encoding genes are silenced^11^. Two repression-associated chromatin modifications are evident: H3K9me3 is abundant within hypomethylated blocks, while H3K27me3 denotes their boundaries^12,13^. Conflicting models have suggested that hypomethylated blocks are either a direct consequence of cell transformation^14^, or an incidental result of excessive cell proliferation^13,15^. The functional consequences of hypomethylation-associated gene silencing, and potential selection pressures that shape such domains, are not well understood. A recent study of advanced colon cancers proposed an intrinsic tumor suppressive mechanism that may counter cell proliferation^13^, although genome-wide hypomethylation is extensive in advanced cancers and may not reveal specific targets contributing to early tumorigenesis.

Prostate cancer is noteworthy for its characteristically slow evolution from precancerous lesions with low levels of cell proliferation to more invasive, and ultimately metastatic malignancy. Localized prostate cancer may be classified as indolent (Gleason score (GS) 6) or clinically significant (GS≥7) based on histological grade, reflecting differences in differentiation, proliferative index, and metastatic potential^16,17^. GS6 tumors are often safely monitored without therapy, while the more aggressive GS7 and higher tumors are resected surgically or treated with radiation in combination with androgen deprivation therapy. GS8–10 denotes poorly differentiated tumors with an adverse prognosis and high propensity for metastasis. Multiple heterogeneous foci of early tumors are often dispersed throughout the prostate gland, complicating bulk molecular characterization and necessitating careful dissection with single-cell analytic strategies. Conversely, advanced metastatic prostate cancer predominantly affects bone, making it difficult to perform biopsies to study disseminated tumor deposits. Circulating tumor cells (CTCs), comprising potential metastatic precursors isolated from the bloodstream, thus enable single-cell analysis of advanced prostate cancer. Immune checkpoint blockade (ICB) is generally ineffective in treating prostate cancer^18–21^, possibly reflecting the stroma-rich, immunosuppressive environment of primary prostate cancer, but tumor cell autonomous mechanisms may also contribute, in both primary and metastatic disease. Epigenetic changes affecting expression of immune regulatory genes and modulating the responsiveness of prostate cancer to immunological therapies have not been characterized.

In addition to their biological significance, cancer-associated methylation changes are of considerable molecular diagnostic interest for blood-based cancer detection. These rely primarily on CpG island-enriched methylation within short DNA fragments (170 bp) circulating in plasma, a fraction of which are tumor-derived (ctDNA)^22–24^. However, among patients with localized prostate cancers, only 11.2% are detectable using plasma CpG island hypermethylation assays^25^, leading us to ask whether the large genomic coverage provided by hypomethylated domains within CTCs may provide complementary information. To address these questions, we first established genome-wide, high-resolution single-cell bisulfite sequencing of hypomethylated domains within individual prostate CTCs from multiple patients and cancer cell lines, identifying 40 core PMDs, shared across metastatic prostate cancers. The timing of DNA hypomethylation during prostate tumorigenesis reveals that core PMDs are hypomethylated as early as indolent GS6 tumors, identifying a single predominant genomic locus, the *CD1A-IFI16* gene cluster, encompassing the entire family of *CD1* lipid antigen presentation genes and multiple interferon-inducible genes implicated in innate immunity. Early hypomethylation-mediated gene silencing points to specific tumorigenic pathways with both biological and diagnostic implications.

## Results

### Identification of shared core PMDs and PMIs across single metastatic prostate cancer cells

**Figure 1.**
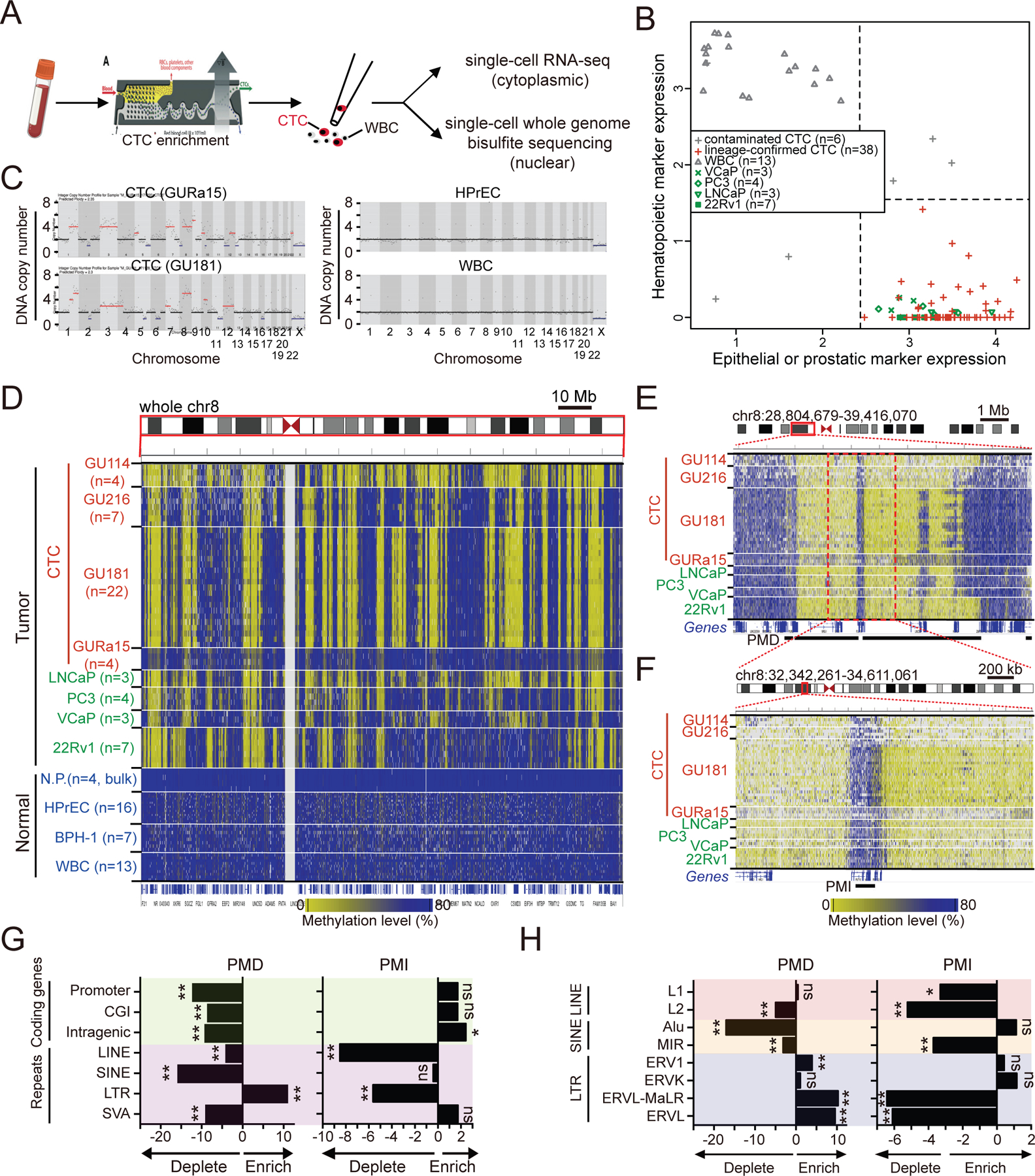
Partially Methylated Domains (PMDs) and Preserved Methylation Islands (PMIs) in single metastatic prostate cancer cells. **(A)** Schematic of CTC enrichment (10^4^-fold leukocyte depletion), and paired DNA methylation sequencing (nucleus) and RNA-seq (cytoplasm) from individual prostate CTCs. **(B)** Confirmation of CTC identity using stringent RNA expression thresholding of prostatic lineage and epithelial *versus* leukocyte markers. Maximum log10 (RPM) expression of epithelial (*KRT7, KRT8, KRT18, KRT19, EPCAM*) and prostatic markers (*AR, KLK3, FOLH1, AMACR*) are plotted against leukocyte markers (*CD45, CD16, CD37, CD53, CD7, CD66b*). Only confirmed CTCs without WBC contamination (red crosses) were used in analyses. **(C)** Representative DNA copy number variation (CNV) analysis in individual CTCs from two patients, compared with a diploid normal prostate epithelial cell (HPrEC) and a healthy donor-derived leukocyte. Single-cell DNA methylation sequencing data was used to infer DNA copy number. **(D)** IGV representation (hg19) of DNA methylation spanning chromosome 8, showing extensive PMDs (yellow) across 37 individual CTCs from four patients (GU114, GU216, GU181 and GURa15), and 17 cells from prostate cancer cell lines (LNCaP, PC3, VCaP, 22Rv1). As controls, 4 normal bulk prostate tissues (N.P.), 36 cells from two prostate epithelial cell lines (HPrEC, BPH-1) and normal leukocytes (WBCs) are shown. Normal methylation level (blue). **(E-F)** Higher resolution of chromosome 8 in IGV, showing precise PMD boundaries shared across individual CTCs and prostate cancer cell lines (panel **E**), with magnified view of the nested PMI, bracketing a few genes, with precise boundaries of preserved methylation flanked by profound hypomethylation (panel **F**). **(G-H)** Components of coding genes and classes of repeats differentially enriched in PMDs *versus* PMIs (panel **G**), with differences among subtypes of repeats (panel **H**). ns, not significant; *P<0.05; **P<0.01, assessed by permutation test.

To characterize genome-wide DNA methylation features of single metastatic prostate cancer cells, we enriched CTCs from five patients with castration-resistant prostate cancer, all with multiple bone metastases and disease refractory to hormonal therapy and performed individual cell micromanipulation and single-cell sequencing^26,27^(Table S1, see Methods). We compared 44 single CTCs with 40 single cells from four prostate cancer cell lines (LNCaP, VCaP, PC3 and 22Rv1) and two non-transformed prostate epithelial cell lines (Human Prostate Epithelial Cells (HPrEC) and Benign Prostate Hypertrophy cells (BPH-1)). HPrECs represent normal prostate epithelium, while BPH-1 cells share luminal cell features with cancer precursors^28–31^. As control for contaminating blood cells within CTC-enriched clinical specimens, we compared single prostate cells with 13 microfluidic-processed single leukocytes (WBCs) from four age-matched healthy men. To confirm the identity of single CTCs, we adapted single-cell multiomics sequencing to enable separation of nucleus from cytoplasm in individual cells, subjecting the former to single-cell whole genome bisulfite sequencing (scBS-seq)^32^ and the latter to single-cell RNA-seq (SMART-seq2)^33^ (Figure 1A, see Methods). On average, we detected 9 million CpG sites for each single-cell DNA methylation sequencing sample, and 5,790 genes (RPM>0) for each single-cell RNA-seq library (Figures S1A and S1B). Transcriptomes of prostate CTCs confirm the expression of expected lineage-specific and epithelial transcripts, and absence of hematopoietic markers (Figures 1B and S1C). Unsupervised hierarchical clustering analysis of all single-cell RNA-seq data reveals three distinct clusters: leukocytes, normal prostate, and prostate cancer (including CTCs and prostate cancer cell lines) (Figure S1D). In addition to transcriptional confirmation, all prostate CTCs demonstrate extensive DNA copy number variations (CNV) inferred from single-cell DNA methylation sequencing (see Methods). These CNV patterns are matched with those inferred from cytoplasmic RNA-seq from the same single cells (Figures 1C, S1E and S1F). As controls, HPrEC cells and WBCs show normal diploid copy numbers (Figure 1C, see Methods). As a final test, principal component analysis (PCA) of promoter methylation patterns readily distinguishes all tumor cells from normal controls (Figure S2A). Taken all together, we applied highly stringent criteria, including both transcriptional and DNA copy number confirmation, to nominate 38/44 (86.4%) initially selected CTCs as bona fide prostate CTCs for detailed single-cell genomic analyses.

We quantified methylation levels of individual cells by binning the genome into 100 kb windows: the methylation distribution of normal cells is unimodal, with a single peak near 80% methylation, whereas virtually all tumor samples exhibit a bimodal distribution, with a varying number of hypomethylated regions (Figures 1D–1F and S2B, see Methods). Overall, DNA hypomethylation constitutes 20–40% of the genome in patient-derived prostate CTCs and prostate cancer cell lines, but <2.5% in normal prostate cells or blood cells (Figure S2C). In contrast to individual CpG islands (CGIs), which often demonstrate focal hypermethylation around gene regulatory regions, the hypomethylated regions in prostate tumor cells span very large gene-poor regions, consistent with previously described PMDs. In total, we identified 1,496 PMDs with a mean size of 1.2 Mb (range 250 kb to 9.2 Mb) across the prostate cancer genome, a number consistent with previous measurements based on bulk tumor sequencing in multiple advanced cancers^8,12,34^ (Figure S2D, Table S2). Notably, on a chromosome-wide view and with the high resolution afforded by single-cell methylation analysis, some PMDs are punctuated by smaller regions, where DNA methylation is retained (Figures 1D–1F). We call these Preserved Methylation Islands (PMIs, see defining criteria in Methods) (Figure S2D, Table S2). In contrast to large gene-poor PMDs, the 1,412 PMIs interspersed within hypomethylated domains are gene-rich, with sharp methylation boundaries that bracket a single gene or a small group of genes (mean PMI size 1.3Mb; range 30.8 kb to 11.1 Mb) (Figures 1F and 1G). The identification of PMIs raises the possibility that selection pressures may preserve methylation, and potentially gene expression, at a small number of genes nested within PMDs.

**Figure 2.**
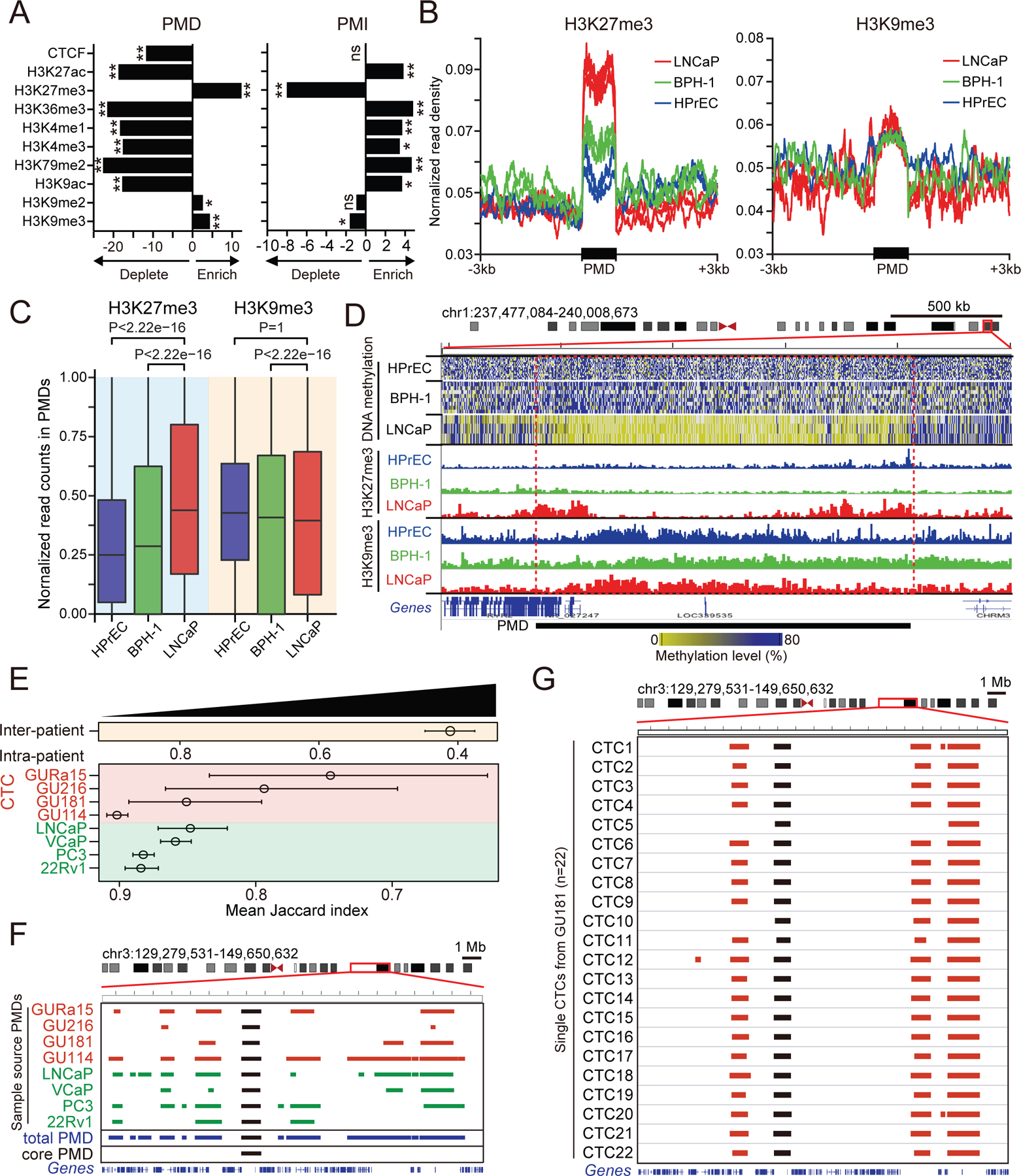
Acquired chromatin marks in prostate cancer PMDs and nomination of shared core PMDs. **(A)** Differential enrichment of chromatin marks within prostate cancer PMDs and PMIs. Annotated chromatin marks from ChIP-seq dataset of PC3 cells in ENCODE (https://www.encodeproject.org/). ns, not significant; *P<0.05; **P<0.01, assessed by permutation test. **(B)** Line plots showing differential enrichment of silencing chromatin marks at PMDs across the genome in prostate cancer cells (LNCaP; 3 biological replicates, red lines), compared with cultured benign prostatic hyperplasia cells (BPH-1; 2 biological replicates, green lines) and normal prostate epithelial cells (HPrEC; 2 biological replicates, blue lines). Across the genome, prostate cancer cells acquire H3K27me3, with highest levels at the boundaries of PMDs (left panel), whereas H3K9me3 enrichment towards the center of PMDs is not altered between cancer and non-transformed prostate cells (right panel). **(C)** Boxplot showing enrichment of Cut and Run signal for H3K27me3, but not H3K9me3, across prostate cancer PMDs between LNCaP cells and non-transformed cell lines (HPrEC and BPH-1). Pvalue, one-tailed Student’s t-test. **(D)** IGV track showing representative cancer-associated PMD (DNA hypomethylation: yellow), with pronounced enrichment of H3K27me3 at PMD borders in cancer cells (LNCaP: red) *versus* non-transformed cells (HPrEC: blue, BPH-1: green), whereas PMD-centered H3K9me3 occupancy is unaltered. **(E)** Inter- and intra-patient heterogeneity of PMDs among single CTCs from four prostate cancer patients (red) and single cells from prostate cancer cell lines. Mean Jaccard index indicates heterogeneity, with higher mean score indicating less heterogeneity among samples. Error bar, mean with 95% confidence interval (CI). **(F-G)** IGV representation of total PMDs and core PMDs at chromosome 3 locus, across 8 sample sources (4 patients and 4 prostate cancer cell lines). Total PMDs (blue) are the union of PMDs defined in each sample source, while core PMDs (black) are shared across all 8 sample sources (panel **F**); representation of PMDs from the single-cell components of an individual sample source (22 CTCs from patient GU181) showing a core PMD shared across all sample sources (black) and neighboring non-core PMDs that are shared by >90% CTCs in this patient, but not across different sample sources (panel **G**). See Figure S2D and Methods for criteria in core PMD and PMI designation.

As demonstrated in other cancers^12,13,35^, PMDs are gene-poor and have strong enrichment of some endogenous retroviral elements (ERVs), notably Long Terminal Repeats (LTRs). In contrast, PMIs in prostate cancer are gene-rich with relative absence of long interspersed nuclear elements (LINEs) and LTRs (Figures 1G and 1H). Previous studies show that PMDs in breast and colon cancers exhibit depletion of active chromatin marks (H3K4me1/3, H3K27ac, H3K36me3) and enrichment of repressive histone modifications, including H3K9me3 at the center of the domains and H3K27me3 at their borders^12,13^. To confirm these chromatin changes in prostate cancer, we used cultured cell lines, to analyze chromatin landscapes using ChIP assays. Analysis of prostate cancer cells (LNCaP and 22Rv1) confirms the differential positioning of repressive H3K9me3 marks at the center and H3K27me3 at the border of hypomethylated domains (Figures 2A, 2B and S3A). However, direct comparison of cancer cells with non-transformed prostate epithelial and basal cells (HPrEC and BPH-1) at the same PMDs indicates that changes associated with malignancy primarily relate to H3K27me3 deposition. Indeed, Cut and Run assays show profound enrichment of H3K27me3 at PMD borders in cancer cells compared with normal cells, whereas central H3K9me3 marks are abundant at these loci, but invariant between normal and cancer cells (Figures 2B–2D and S3A–S3C). Thus, hypomethylation-associated gene silencing in cancer cells is primarily correlated with the acquisition of H3K27me3 histone modification flanking these chromosomal domains. In contrast, genes within PMIs show strong enrichment for activation (H3K4me1/3, H3K27ac and H3K36me3) and absence of repression (H3K27me3 and H3K9me2/3) (Figure 2A).

At the single-cell level, both PMDs and PMIs show substantial intra-patient and inter-patient heterogeneity (Figures 2E–2G and S3D–S3F), leading us to define common domains shared across all single prostate cancer cells that may identify common and hence functionally significant pathways. Of 1,496 PMDs, only 40 (2.7%) are universally hypomethylated, with the mean quantile normalized methylation level <25%, across cells from all four patients with metastatic prostate cancer and four prostate cancer cell lines (Figure S2D, Table S2, see Methods). The 40 core PMDs have a mean size of 2.5 Mb (range 353.4 kb to 7.7 Mb) and encompass 143 protein-encoding genes, a gene density of 1.44 gene/Mb. Hypomethylation associated with cell proliferation is thought to be more rapid in loci that have reduced CpG content^15,36^. Indeed, we note that the core prostate PMDs exhibit reduced CpG residue content, compared with other PMDs across the genome (P<0.0066, Figure S3G), providing a possible explanation for their universal hypomethylation. In the same single prostate cancer cells, analysis of the 1,412 PMIs for intersection across all prostate cancer patients and prostate cancer cell lines identifies 44 core PMIs (Figure S2D, Table S2, see Methods). Core PMIs have a mean size of 371.7 kb (range 27 kb to 1.9 Mb) and harbor 255 protein-encoding genes, with a gene density of 15.6 genes/Mb (Figure S3H).

Our single-cell analysis of prostate cancer cells identifies a small fraction of PMDs that are universally shared, which we describe as core PMDs, and it also reveals that interspersed within these large PMDs are small gene-rich islands with preserved DNA methylation, that we call PMIs.

### Hypomethylation of core PMDs is an early event in prostate tumorigenesis

**Figure 3.**
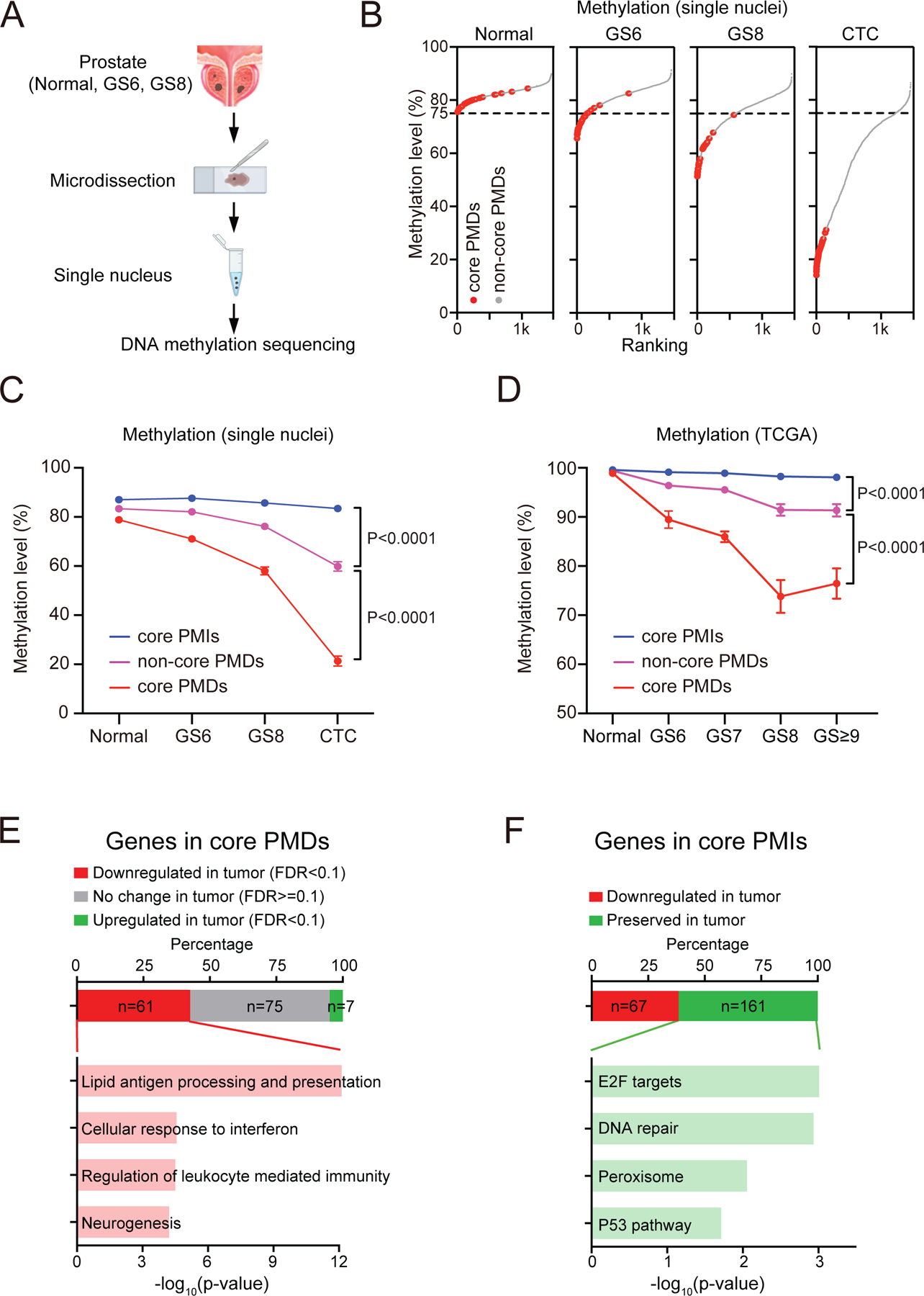
Demethylation of core PMDs during early prostate tumorigenesis suppresses immune-related genes, while core PMIs spare proliferation genes. **(A)** Schematic showing prostate tumor microdissection, single nucleus isolation and single-cell DNA methylation sequencing. **(B)** Ranking of methylation level at 40 core PMDs (red dots) among all 1,496 total PMDs, as a function of timeline from normal prostate, to localized (GS6; GS8) and metastatic cancer (CTCs), showing early demethylation of core PMDs. Within normal prostate, all 40 core PMDs have methylation level >75%, and 31 are hypomethylated as early as GS6. **(C)** Quantitation of demethylation as a function of Gleason Score (GS). Demethylation of core PMDs (red curve) precedes that of other PMDs (magenta) within microdissected prostate tumor cells and in CTCs. In contrast, core PMIs nested between PMDs (blue) show minimal DNA methylation changes during tumorigenesis. Error bar, mean with SEM. Statistical analysis of DNA methylation curves utilizing longitudinal linear mixed effects model, by which tumor progression x methylation domains was tested. **(D)** Quantitation of demethylation as a function of GS in TCGA prostate cancer methylation array data, showing early and progressive loss of methylation of core PMDs (red curve), with an attenuated trend for other PMDs (magenta). The core PMIs (blue) display stable DNA methylation pattern during prostate tumorigenesis. Statistical analysis as for panel C. **(E-F)** Gene set enrichment analysis (GSEA) of genes residing within core PMDs and downregulated in primary prostate cancer **(E)**, and of genes residing within core PMIs with gene expression preserved (up-regulated and not significantly changed) in primary prostate cancer **(F)**, compared with normal prostate. (FDR <0.1; two-tailed Student’s t-test with FDR correction).

DNA hypomethylation progresses during cancer evolution to ultimately encompass large regions of the non-coding and gene-poor genome within advanced cancers^37^. By analogy with early genetic driver mutations, however, non-random epigenetic silencing may play an important role in initiating tumorigenesis, with selection pressures guiding recurrent early events. Having defined core PMDs shared across single metastatic prostate cancer cells, we sought to identify genomic loci that are consistently subject to early silencing during tumorigenesis. Given the characteristic admixture of tumor and stromal cells in localized prostate cancer, we obtained frozen tissue sections from prostatectomy specimens and purified single nuclei for molecular analysis. Tumor origin of individual nuclei was confirmed by CNV inferred from whole genome bisulfite sequencing, and we computed a CNV score (absolute DNA copy number changes per Mb) to complement Gleason histological scoring, as an independent measure of tumor progression (Figure 3A, see Methods). In addition to Gleason histological scoring of localized prostate cancer, we computed a CNV score (absolute DNA copy number changes per Mb) to quantify genomic instability in single nuclei from different prostatectomy samples, as an independent measure of tumor progression. In total, we profiled 38 primary tumor nuclei from five patients with low grade (GS6) prostate cancer, 62 nuclei from another five patients with high grade (GS≥8) disease, and 78 normal prostate cells from adjacent tissue sections, comparing these with the 38 CTCs from patients with metastatic disease (Table S1). Inferred CNV from our high resolution single nucleus analysis identifies Chr8p loss (containing *NKX3–1*, *BMP1*, *FGFR1* genes and multiple microRNAs) as one of the earliest genetic events in prostate tumorigenesis, shared by >43% of cancer cells in GS6 tumors (Figure S4A). Early allelic loss of this locus has been reported in prostate cancer^38–41^. Interestingly, GS6 prostate cancer cells with Chr8p loss show more hypomethylation across PMDs, pointing to coordinated early timing of CNV and hypomethylation (Figure S4B). At the single-cell level across different tumors, hypomethylation at prostate PMDs exhibits less heterogeneity than do hypermethylated CpG promoter regions (Figures S4C and S4D).

Remarkably, core PMDs initially defined by their universal hypomethylation in metastatic prostate cancer cells show profound enrichment at the earliest stages of tumorigenesis. In early GS6 tumors, 77.5% (31/40) core PMDs are hypomethylated, compared with only 8% (115/1,456) of non-core PMDs (Figure 3B). Indeed, mean quantitative methylation levels within core PMDs decline from 78.4% (normal prostate), to 70.4% (GS6), 57.2% (GS8), and 20.2% in metastatic CTCs. Comparable methylation levels across all prostate PMDs decline more slowly: 82.2% (normal prostate), 80.9% (GS6), 74.7% (GS8) and 57.6% (CTCs) (Figure 3C). By contrast, methylation at interspersed core PMIs shows little change from normal prostate nuclei to GS6, GS8, and metastatic prostate CTCs. Compared with hypomethylation of large chromosomal domains, focal hypermethylation of CpG islands within gene regulatory regions increases gradually from 27.5% (normal prostate), to 30.7% (GS6), 31.9% (GS8), and 34.3% (CTCs) (Figure S4E), as does aneuploidy measured by CNV score (Figure S4F). We observed no confounding correlation (FDR>0.1) between CNV and DNA methylation for core PMDs (Figure S4G). Our observations of accelerated progressive demethylation of core PMDs in early prostate cancer are confirmed by analysis of TCGA prostate cancer methylation array data stratified by Gleason Score (Figure 3D), as well as whole genome bisufite sequencing in primary and metastatic prostate tumors^34,42^ (Figure S4H). Core PMIs show preserved methylation patterns independent of Gleason Score (Figures 3D and S4H).

Taken together, core PMDs begin to lose DNA methylation within indolent GS6 prostate cancers, one of the earliest identifiable lesions in prostate tumorigenesis. This early timing explains their universal hypomethylation in advanced cancers, compared with more heterogeneous hypomethylation domains that emerge during subsequent tumor progression.

### Silencing of immune-related genes within core PMDs and persistent expression of proliferative genes within PMIs

To address the functional consequences of early DNA hypomethylation, we identified protein-encoding genes localized to core PMDs that display loss of expression across the large prostate cancer TCGA database^39^. Among the 143 protein-coding genes residing within the 40 core PMDs, 68 (48%) are consistently and significantly differentially expressed between normal prostate and primary prostate tumors, with 61 (90%) suppressed and 7 (10%) induced in cancer. Remarkably, 12/61 (20%) silenced genes within core prostate PMDs are immune-related. GSEA analysis reveals lipid antigen processing and presentation (P<1.96E-13) and cellular response to interferon (P<2.74E-5) as the two most highly enriched pathways (Figure 3E, see Methods). Conversely, of the 255 protein-encoding genes within the 44 core PMIs, 161 (63.1%) are comparably expressed in prostate cancer and normal prostate tissues in the same TCGA database. The top GSEA pathways all relate to cell proliferation, including E2F targets (P<0.000975) and DNA repair (P<0.00116) (Figures 3F, S4I–J). As control, GSEA pathway analysis does not identify statistically significant enrichment among core PMD-derived genes that are not expressed or not silenced in prostate cancer, or among core PMI-derived genes without preserved expression. Thus, identifying early and consistent changes in DNA methylation in prostate cancer cells points to silencing of immune-related genes, with selective sparing of genes encoding proliferative drivers, as initial steps in prostate tumorigenesis.

### PMD-associated silencing of the CD1A-IFI16 gene cluster

**Figure 4.**
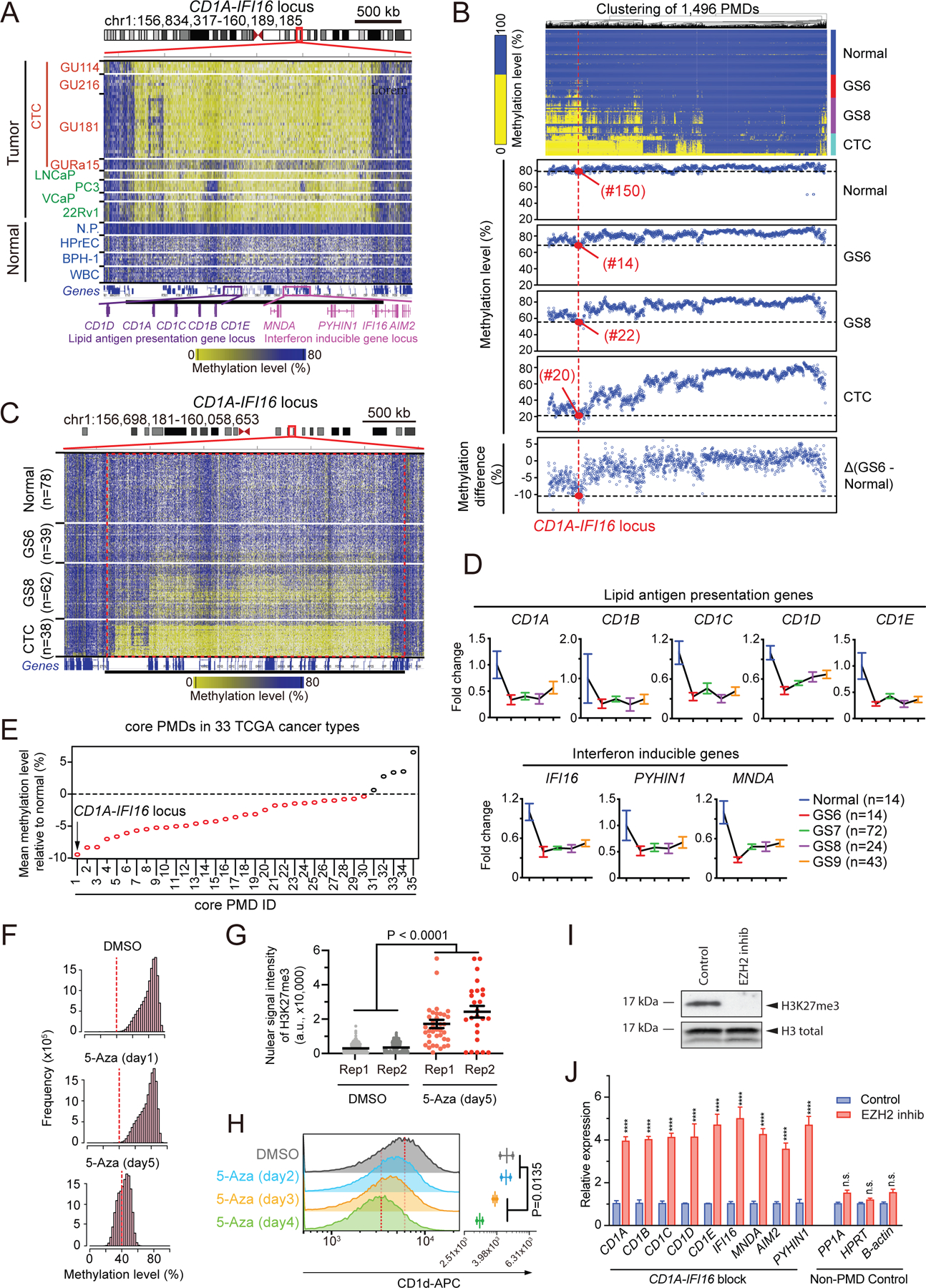
Correlation of DNA demethylation at the *CD1A-IFI16* locus with accumulation of chromatin silencing marks and reduced gene expression. **(A)** IGV of single-cell DNA methylation at the *CD1A-IF16* genomic locus, including five lipid antigen presentation and four interferon inducible genes. Tumor cells (37 single CTCs from four prostate cancer patients (red) and 17 single cells from four prostate cancer cell lines (green)) exhibit marked hypomethylation at this locus (shaded yellow), while normal samples (4 bulk normal prostate tissues, 37 single cells from normal prostate cell lines and leukocytes (blue)) show a preserved DNA methylation (shaded blue). **(B)** Heatmap (upper panel; hypomethylation shaded yellow) and matched quantitative scatter plots (lower panel) of single-cell DNA methylation levels within all 1,496 prostate cancer PMDs, showing progression from normal prostate to localized prostate cancer (GS6, GS8) and metastatic CTCs. The *CD1A-IFI16* locus (dashed vertical red line) shows early and profound demethylation, starting at GS6, with its rank number across all PMDs at each tumor stage shown in parentheses (red). **(C)** IGV screenshot of single-cell DNA methylation data showing progressive demethylation of *CD1A-IFI16* locus (box with red dashed line) from normal prostate cells to localized (GS6 and GS8) and metastatic prostate cancer (CTCs). Heterogeneity of hypomethylation (shaded yellow) across single cells is evident at GS6, becoming more prevalent at GS8, and uniform in CTCs . **(D)** Plots showing suppressed expression of lipid antigen presentation and interferon inducible genes within the *CD1A-IFI16* locus, during transition from normal prostate to low-grade GS6, with persistent silencing in higher grade GS7, 8 and 9 cancers (TCGA dataset). Error bar, mean with SEM. **(E)** Analysis of 33 different tumor types (TCGA) for DNA methylation differences at core prostate cancer PMDs, compared with corresponding normal tissues. 30 of 35 (86%) evaluable PMDs are hypomethylated across all tumor types (red circles), with the *CD1A-IFI16* locus having the strongest hypomethylation. **(F)** Histograms of DNA methylation level within 100kb windows (200bp offsets) across the genome in normal prostate cells (BPH-1), following 5-azacytidine treatment (days 1 and 5), compared with DMSO control. **(G)** Quantitation of H3K27me3-related fluorescence intensity within single-cell nuclei (confocal microscopy). Error bar, mean with SEM. P-value, two-tailed Student’s t-test. **(H)** Sequential reduction in CD1d protein expression in normal prostate cells (BPH-1) treated with 5-azacytidine, compared with DMSO control. Representative flow cytometry (left panel); median fluorescence intensity (right panel). Error bar, mean with SEM. P-value, two tailed Student’s t-test. **(I-J)** Western blot showing reduced H3K27 trimethylation in 22Rv1 cells treated with EZH2 inhibitor GSK126 for 6 days (panel **H**); qPCR of genes within the *CD1A-IFI16* cluster show induced expression (panel **I**), while non-PMD resident control genes (*PP1A, HPRT* and ***β****-actin*) remain unchanged. P-value, Tukey’s multiple comparison tests, where GSK126 treatment conditions (red bars) were compared to controls (blue bar). n.s. not significant; ****P<0.0001.

A remarkable feature of core PMD-associated gene silencing is targeting of the entire *CD1* family of lipid antigen presentation genes (*CD1A, CD1B, CD1C, CD1D* and *CD1E*) and four interferon inducible genes of the Pyrin and HIN domain (PYHIN) family involved in immune sensing of non-self DNA (*IFI16, AIM2, PYHIN1* and *MNDA*). These genes are clustered within the same core hypomethylation block at chromosome 1q23.1 (hereafter, *CD1A-IFI16* block), consistent with a single genomic locus playing a major role in integrating these two immune recognition pathways (Figure 4A). The *CD1* gene family encodes MHC class I-like molecules that exclusively present non-peptides (e.g. glycolipids) to Natural Killer-T (NKT) cells, a rare subset of T cells implicated in both innate and adaptive immunity^43–45^. The CD1 pathway is primarily implicated in innate immunity to infectious agents, although a possible role for lipid antigens in anti-tumor immunity is also postulated^46,47^. Among interferon-inducible genes, *IFI16* is highly expressed in normal prostate cells: it is reported to bind non-self dsDNA in both nucleus and cytoplasm in a DNA length-dependent manner, recruiting STING and further activating interferon signaling^48^.

DNA methylation of the *CD1A-IFI16* locus declines early and rapidly, scoring as the 14^th^ earliest across all genome-wide PMDs measured at GS6 (Figure 4B). Heterogeneity in hypomethylation at *CD1A-IFI16* is evident within single prostate cancer cells at early stage GS6 tumors, progressively increasing in both fraction of tumor cells and degree of hypomethylation within individual tumor cells as they evolve to GS8 and ultimately to metastatic CTCs (Figures 4C and S5A). This early and progressive loss of DNA methylation at the *CD1A-IFI16* locus, compared with the slower rate of demethylation genomewide, is also evident in analysis of public databases of primary and metastatic prostate cancer^34,42^ (Figure S5B). Analysis of TCGA prostate cancer data stratified by Gleason Score further confirms early progressive loss of methylation within the *CD1A-IFI16* locus (Figure S5C), and the associated transcriptional downregulation of the encoded genes as early as GS6 tumors (Figures 4D). The accelerated decline in DNA methylation at *CD1A-IFI16* is not driven by gene copy number changes, as confirmed by comparing single nuclei with or without CNV at this locus (Figure S5D).

Early DNA hypomethylation at the *CD1A-IFI16* locus is not restricted to prostate cancer. DNA methylation datasets at defined stages of cancer progression are available for both colon and thyroid cancers^9^, both of which demonstrate earlier and more progressive demethylation of *CD1A-IFI16*, when compared to other core PMDs (Figure S5E). Furthermore, analysis of methylation profiles in a TCGA cohort including more than 1,000 samples spanning 33 cancer types (https://portal.gdc.cancer.gov) identifies the *CD1A-IFI16* locus as consistently hypomethylated in 23 different cancers (Figures S5F–G). Across all 33 cancer types, *CD1A-IFI16* demonstrates the greatest degree of DNA hypomethylation compared with all other core PMDs (Figure 4E), and 19 of the 33 cancers show a significant correlation between hypomethylation of this locus and reduced RNA expression of *CD1A-IFI16* resident genes (Figure S5H). Early and profound DNA hypomethylation at *CD1A-IFI16* is thus a consistent feature across multiple cancers.

Along with DNA hypomethylation of the *CD1A-IFI16* locus, we observed the expected enrichment for H3K27me3 chromatin marks, comparing prostate cancer *versus* normal prostate cell lines, together with suppression of the encoded genes within that locus (Figures S6A, B and S6C, Table S3). Extending this analysis to nuclei from microdissected GS6 and GS8 tumors using ultra-low-input native ChIP-seq (ULI-NChIP), we observe marked progressive enrichment of H3K27me3 at the *CD1A-IFI16* locus in early GS6 tumors compared with normal prostate epithelium (Figures S6D–E), whereas other PMDs show increased H3K27me3 only at GS8 (Figure S6F). Finally, within high purity TCGA prostate samples (tumor purity >0.5 inferred by ABSOLUTE algorithm), all five lipid antigen presentation genes and three of the four PYHIN interferon inducible genes are suppressed in primary prostate tumors (n=188) compared with normal prostate (n=14) (Figure S6G). The suppression of *CD1A-IFI16* gene expression is observed at the earliest timepoint of DNA hypomethylation (GS6), and it persists as DNA hypomethylation progresses, suggesting a potential threshold effect. Thus, immune-related genes within the *CD1A-IFI16* cluster are among the earliest targets of cancer hypomethylation-induced transcriptional silencing.

### Functional recapitulation of hypomethylation-associated silencing at CD1A-IFI16 locus

To investigate the functional relationship between DNA methylation, repressive chromatin marks and expression of PMD-resident genes, we applied the DNA demethylating agent 5-azacytidine (5 μM) to the human prostate epithelial cells (BPH-1), in which the *CD1A-IFI16* locus shows normal DNA methylation levels (Figure 4A). Global DNA methylation declines by 4.9 % after 24 hrs of 5-azacytidine, and by 37.7% after 5 days of drug exposure, compared with DMSO controls (Figure 4F), with the *CD1A-IFI16* locus showing progressive DNA demethylation upon 5-azacytidine treatment (Figure S7A). Bisulfite treatment and Sanger sequencing confirms gradual demethylation at *CD1A-IFI16* (DMSO: 75.9%, day5: 40.8%) (Figure S7B). Ectopically-induced demethylation is accompanied by marked increase of the chromatin silencing mark H3K27me3, as shown by quantitative imaging of nuclei (7.18-fold increase after 5 days) (Figures 4G and S7C), along with H3K9me3 (Figures S7D–E), and associated with reduced expression of *CD1* (Figure 4H). Thus, DNA hypomethylation appears to trigger the recruitment of chromatin suppressive marks at the *CD1A-IFI16* locus, along with repression of the resident genes.

We then tested the converse model, using an inhibitor of the EZH2 methyltransferase, GSK126, to suppress H3K27me3 in prostate cancer cells, in which the *CD1A-IFI16* locus is hypomethylated and silenced. Treatment of three prostate cancer cell lines (22Rv1, LNCaP and VCaP) with GSK126 results in loss of global H3K27 trimethylation, associated with a dramatic increase in expression of all the genes within the *CD1A-IFI16* locus (Figures 4I–J and S7F–G). Together, these observations further support the role of chromatin silencing marks in repressing coding genes within the *CD1A-IFI16* locus and other PMDs.

### Re-expression of lipid antigen presentation or interferon-inducible genes restores anti-tumor immunity in a mouse model

**Figure 5.**
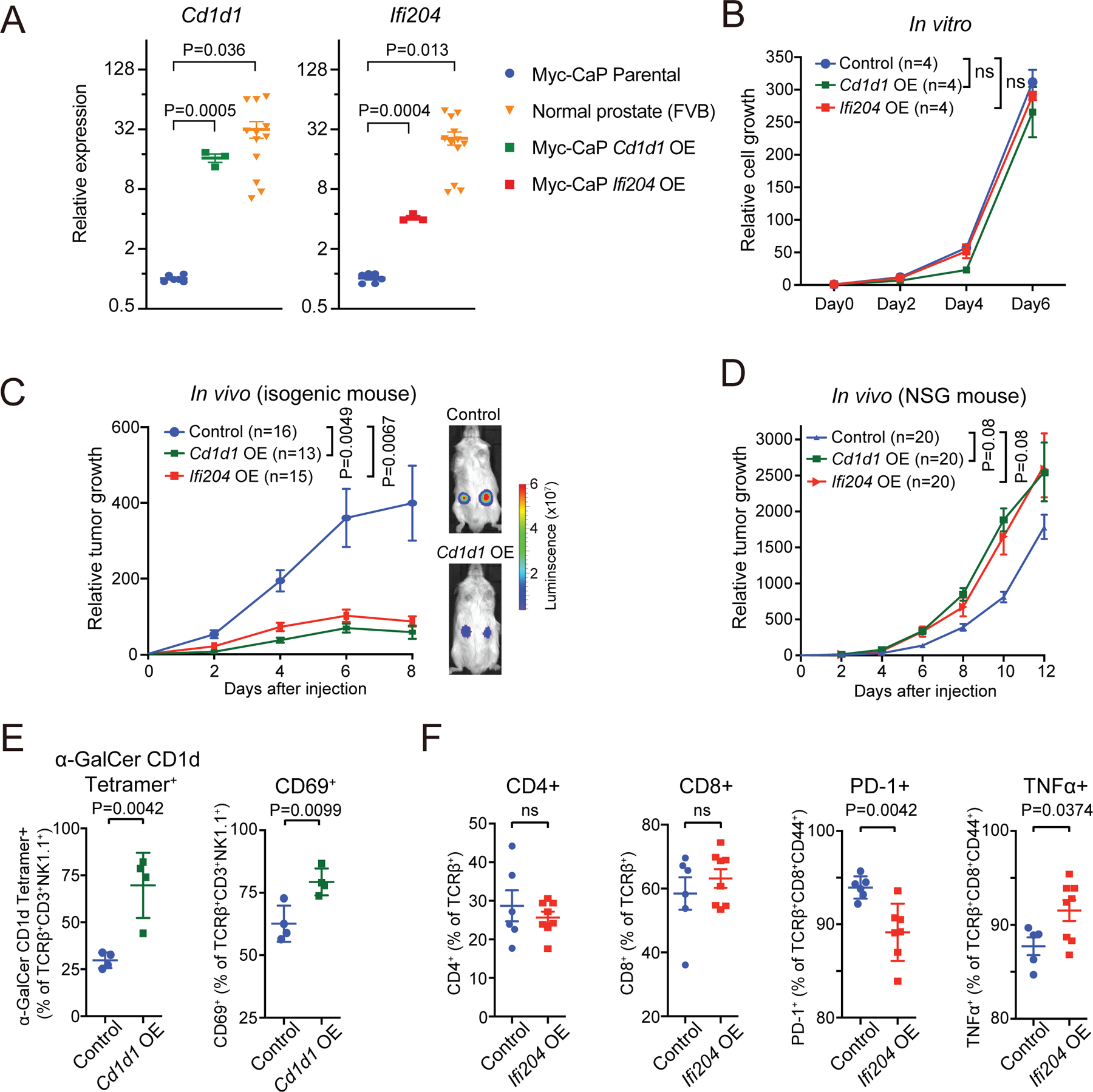
Restoring expression of genes within *CD1A-IFI16* syntenic locus abrogates tumorigenesis in an immunocompetent mouse prostate cancer model. **(A)** Plots quantifying *Cd1d1* and *Ifi204* mRNA in the murine prostate tumor cell line Myc-CaP, which have silenced the syntenic genes (blue), compared to normal prostate cells from 4 isogenic mice FVB (orange). Ectopic expression of murine *Cd1d1* (*CD1D* ortholog, green) and *Ifi204* (*IFI16* ortholog, red) is comparable to that of normal prostate. Error bar, mean with SEM. **(B)** Overexpression (OE) of *Cd1d1* or *Ifi204* in Myc-CaP cells does not alter *in vitro* proliferation compared with controls. Error bar, mean with SD. **(C)** Overexpression of either *Cd1d1* (green) or *Ifi204* (red) in Myc-CaP cells (mCherry-luciferase tagged) suppresses tumorigenesis in isogenic immunocompetent FVB mice. Mock-transfected control tumors are shown as control (blue). Tumor size quantified by luciferase imaging (representative images). Error bar, mean with SEM. **(D)** Myc-CaP cells engineered as in (C) show no difference in tumor growth in immune-deficient NSG mice. Error bar, mean with SEM. **(E)** Flow cytometry of *Cd1d*-restored Myc-CaP tumors in FVB mice, showing recruitment of CD1d-restricted NKT cells (marked by α-GalCer CD1d Tetramer) and activated NKT cells (marked by CD69), compared with controls. Error bar, mean with SD. **(F)** Flow cytometry of *Ifi204*-restored Myc-CaP tumors in FVB mice, showing unaltered infiltration of total CD4^+^ and CD8^+^ T cells, but reduced immune infiltration by PD-1^+^ CD8^+^ T cells and increased presence of TNFα^+^ CD8^+^ T cells, compared with controls. Error bar, mean with SD. P-values, two-tailed Student’s t-test; ns, not significant.

To explore the potential significance of *CD1A-IFI16* silencing, we tested the consequences of restored expression in a murine model of early prostate tumorigenesis. The mouse prostate cancer cell line Myc-CaP is derived from a genetically engineered model with prostate-specific expression of a c*-Myc* transgene driving androgen-dependent tumorigenesis^49^. Single-cell methylation sequencing of Myc-CaP cells shows uniform hypomethylation of two chromosomal loci syntenic with the single human *CD1A-IFI16* locus, and encompassing the two murine lipid antigen presentation genes (*Cd1d1* and *Cd1d2*) and the orthologous PYHIN interferon inducible genes (*Ifi204, Aim2, Pyhin1* and *Mnda*), respectively (Figures S8A and S8B). Repressive H3K27me3 and H3K9me3 marks are enriched at the *Cd1d* and interferon inducible genes (Figures S8A and S8B). The major *CD1* murine ortholog *Cd1d1* and the *IFI16* murine ortholog *Ifi204* are repressed in Myc-CaP tumor cells, compared with normal prostate tissues dissected from isogenic FVB mice (Figure 5A). We ectopically expressed *Cd1d1* (16.1-fold) or *Ifi204* (4.1-fold) in Myc-CaP cells by lentiviral transduction, achieving levels comparable to those of normal mouse prostate (Figure 5A and Table S3, see Methods). Cell surface localization of restored Cd1d1 is evident using both flow cytometry and confocal microscopy (Figures S8C and S8D).

Ectopic expression of *Cd1d1 in* Myc-CaP cells does not alter proliferation *in vitro*, but these cells fail to produce tumors in isogenic immune competent FVB mice, when inoculated either subcutaneously or by direct intraprostatic injection (Figures 5B, 5C and S8E). This effect is dependent upon immune cell activation, since inoculation of the same *Cd1d1-*expressing Myc-CaP cells into immunodeficient NSG mice does not suppress their ability to give rise to primary tumors (Figure 5D). *Cd1d* specifically mediates the presentation and activation of lipogenic antigens to NKT cells, a rare T cell subpopulation expressing *Cd40lg* and *Icos* (http://rstats.immgen.org/Skyline/skyline.html)^50^, and tumors from *Cd1d1*-restored Myc-CaP cells in FVB immune competent mice show increased expression of *Cd40lg* (2.8-fold; P=0.0063) and *Icos* (3.2-fold; P=0.00023) compared with controls (Figure S8F and Table S3). Flow cytometric analysis of tumor immune infiltrates in *Cd1d1*-restored tumors indicates more abundant Cd1d-restricted NKT cells (P=0.0042), along with increased binding to the high affinity synthetic NKT cell ligand alpha-Galactosyl Ceramide (α-GalCer) tetramer and an increase in the CD69 marker of NKT cell activation (P=0.0099) (Figures 5E and S9A–C). To test the consequences of restored *Cd1d1* expression in another mouse isogenic tumor model, we restored its expression in the LLC-1 lung epidermoid carcinoma model, which does not express *Cd1d1*. Ectopic expression of *Cd1d1* in LLC-1 reduces tumor growth upon subcutaneous inoculation into immune competent isogenic C57BL/6 mice, despite unaltered *in vitro* proliferation (Figures S8G–J).

We then tested the effect of restored expression in Myc-CaP cells of *Ifi204,* the murine ortholog of the interferon inducible gene *IFI16*. Re-expression *Ifi204* also suppresses Myc-CaP tumorigenesis in immune competent FVB mice, without any anti-proliferative effect *in vitro* (Figures 5B and 5C). This effect is not evident in immune deficient NSG mice, pointing to an immunological effect (Figure 5D). *T*umors derived from *Ifi204*-expressing Myc-CaP cells in FVB mice show no difference in the total number of CD4^+^, CD8^+^ T cells or in the expression of general marker of T cell activation (Figures 5F, S9D and S9E). However, compared to parental controls, *Ifi204*-reconstituted tumors have a dramatic reduction in expression of the co-inhibitory receptor PD-1 within CD8^+^ T cells (P=0.00042), along with an increase in the functional intracellular cytokine TNFα (P=0.0374), all consistent with activated CD8^+^ T cell cytotoxic function (Figures 5F, S9F and S9G). No change is evident in expression of other co-inhibitory receptors (TIGIT, LAG3 or TIM3) or cytokine (IFNγ) in CD8^+^ T cells (Figures S9H–S9K).

Thus, ectopically restored expression of either *CD1* or *IFI16* murine orthologs in cancer cells with DNA hypomethylation-induced silencing suppresses tumor formation, a finding only evident in immune competent mice, and associated with evidence of selectively increased anti-tumor activity. Our results indicate that at least two distinct immune populations are impaired by silencing of the *CD1A-IFI16* locus (NKT cells modulated by *Cd1d1* and cytotoxic CD8+ T cells affected by *Ifi204*), suggesting a complex immune-modulatory function of this multigene locus in tumorigenesis.

### Detection of CTC-derived DNA hypomethylation in blood specimens using Nanopore sequencing

**Figure 6.**
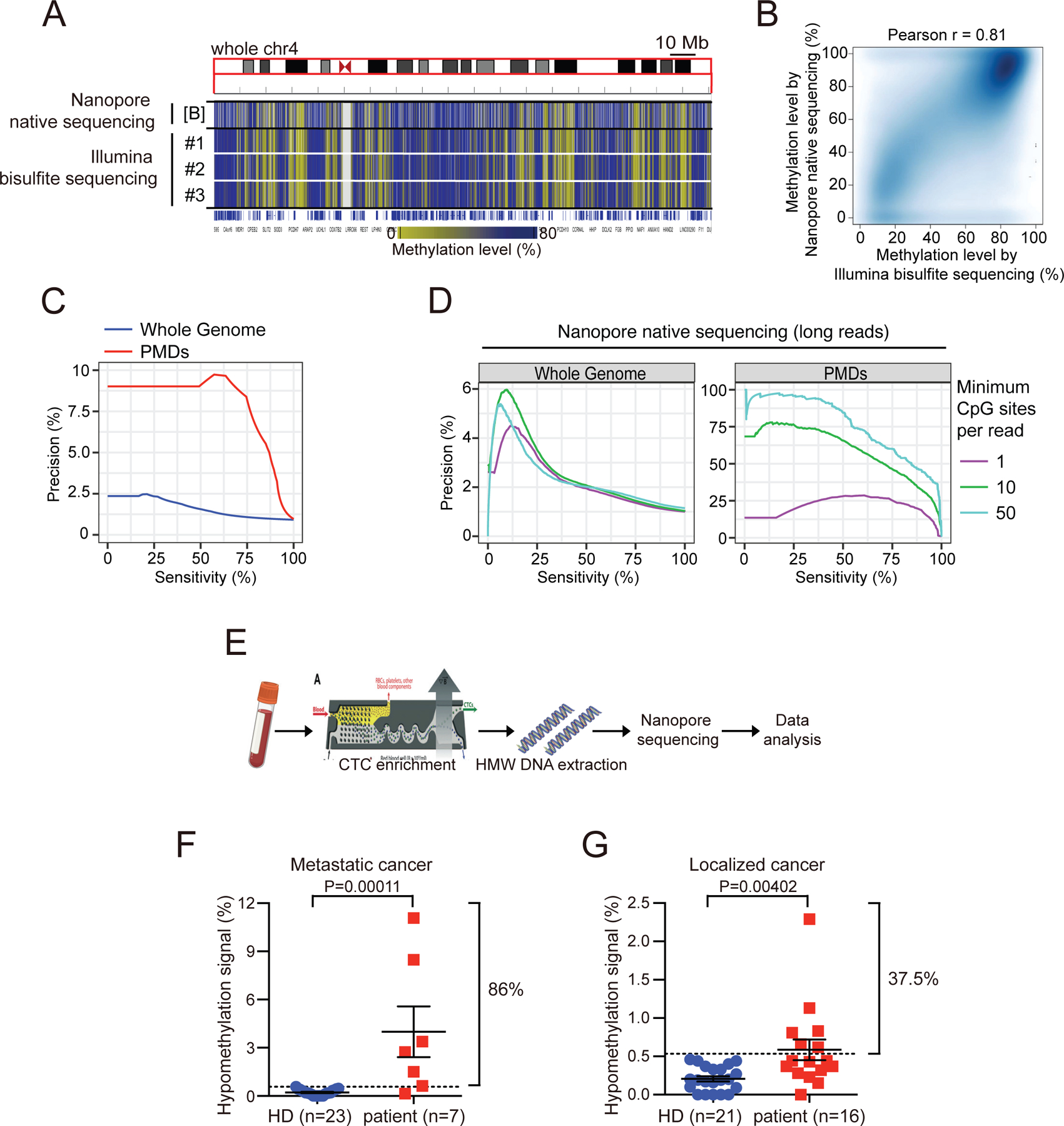
Detection of CTC-derived DNA hypomethylation in blood specimens using Nanopore sequencing. **(A)** IGV screenshot showing concordance of DNA hypomethylation measurements between Oxford Nanopore native sequencing of bulk VCaP cells [B], compared with Illumina bisulfite sequencing of three single VCaP cells (#1, #2, #3). DNA methylation across entire chromosome 4 is shown (hypomethylation in shaded yellow). **(B)** Scatter plot showing high Pearson correlation (r=0.81) between Nanopore native sequencing and Illumina bisulfite sequencing. **(C-D)** Mathematical modeling showing minimal precision using short reads (average 5 CpG sites per read) for detection of hypomethylated DNA domains. Modest improvement in detection is provided by interrogating predetermined PMDs, instead of whole genome (panel **C**). Significantly improved precision is predicted using Nanopore long read sequencing (10 or 50 CpGs per read). Highest predicted accuracy by combining Nanopore long reads (>10 CpG sites per read) with selected analysis-predetermined PMD regions (panel **D**). **(E)** Schematic of microfluidic CTC enrichment (followed by direct Nanopore sequencing of bulk cells (approximatly 0.1% CTC purity). HMW, high molecular weight. **(F-G)** Scatter plot quantitation of hypomethylation signal by Nanopore sequencing, comparing leukocyte-depleted blood samples from patients with either metastatic (panel **F**) or localized prostate cancer before surgical resection or radiation therapy (panel **G**), *versus* healthy age-matched male donors (HDs). Error bar denotes mean with SEM. P-value assessed by two-tailed Student’s t-test. Dotted lines indicate thresholds of hypomethylation signal that encompass all healthy donors tested, with the fraction of cancer patients with hypomethylation signal above that threshold considered positive.

While our study was focused on the characterization of early methylation changes in prostate tumorigenesis and their potential biological consequences, we also note the recent application of CpG island hypermethylation as a blood-based diagnostic assay for early cancer detection^24,25^. Genome-wide screening for changes in DNA methylation may be more sensitive than mutation-based assays, particularly in tumors like prostate cancers, which do not harbor well defined recurrent driver mutations. Nonetheless among all cancers tested, early prostate cancer shows one of the lowest detection rates (11.2%), using screening for CpG island hypermethylation^25^. CTCs are shed into the blood by invasive localized prostate cancers long before they establish metastases^51–53^, raising the possibility that they may provide an orthogonal assay for early cancer detection. Given the specificity of DNA hypomethylation domains in cancer cells and their large genomic size, we reasoned that they may provide high sensitivity and quantitative signal for cancer detection, following CTC enrichment in blood specimens. For such blood-based rare cell signal detection studies, we applied a screen for all prostate PMDs, rather than the much smaller number of core PMDs, so as to increase coverage to a large fraction of the prostate cancer genome. Oxford Nanopore long-read native sequencing typically produces sequencing reads up to 100 kb, and directly identifies methylated CpG residues (5mC), without requiring bisulfite conversion in library preparation^54,55^. In its current configuration, Nanopore signal analysis does not readily identify 5-hydroxymethyl cytosines (5hmC), which are considerably less abundant than 5mC, and are also not distinguished from 5mC in conventional bisulfite sequencing. Indeed, Nanopore sequencing of the VCaP prostate cancer cell line clearly defines DNA hypomethylation domains, which faithfully recapitulate those identified in these cells using standard bisulfite sequencing (Figures 6A and 6B).In contrast to the short Illumina sequencing reads (usually harboring <5 CpG sites per read), mathematical modeling indicates that the long reads generated by Nanopore sequencing would empower detection with significantly higher precision for rare signal (Figures 6C and 6D, see Methods). We therefore processed 10 ml blood specimens from patients with either localized or metastatic prostate cancer, using microfluidic enrichment to deplete leukocytes (10^4^-fold depletion), but without further CTC purification or individual CTC micromanipulation (Figure 6E, see Methods). While 23 age-matched healthy donors (HDs) show minimal DNA hypomethylation signal (<0.6%), 6 out of 7 (86%) patients with metastatic prostate cancer have significant signal (from 0.62% to 11.08% of sequencing reads, P=0.00011), as do 6/16 (37.5%) patients with localized prostate cancer (from 0.62% to 2.29% of sequencing reads, P=0.004) (Figures 6F and 6G,Tables S4 and S5). Thus, long-range hypomethylated domains are universal characteristics of prostate cancer and they are detectable from rare CTCs in patient-derived blood specimens. The simplicity and cost effectiveness of Nanopore sequencing raises the possibility of hypomethylation-based cancer detection.

## Discussion

Using single-cell DNA methylation analysis, ranging from indolent low grade localized prostate cancer to metastatic CTCs, we annotated at high resolution the shared hypomethylation domains that constitute core PMDs, along with interspersed islands with preserved methylation, that we identify here as PMIs. PMDs are known to be associated with the peripheral and transcriptionally silenced B compartment of the nucleus^13,56^, raising the possibility that PMIs loop into the active A compartment regions, and hence are spatially distinct from the surrounding silenced chromatin. Given intercellular heterogeneity, the denotation of core PMDs was derived from the intersection of PMDs across many single cells from multiple independent prostate cancers. However, these core PMDs also stand out by virtue of their detection in the earliest low grade prostate cancers (GS6), leading to the suggestion that they are driven by early selective pressures in tumorigenesis, and explaining their universal silencing in advanced prostate cancers. Indeed, silencing within core hypomethylation domains appear to target immune-related genes, including a single chromosomal locus containing the entire family of *CD1* genes and a cluster of interferon-inducible genes. PMIs, in contrast, preserve expression of proliferation-associated genes implicated in cell-cycle and DNA damage repair pathways. DNA methylation changes may thus convey a selective advantage in prostate cancer development, suppressing expression of genes contributing to immune surveillance of nascent tumors, while shielding neighboring genes that enhance cell proliferation. Such selective pressures could drive the very early targeting of the immune-rich *CD1A-IFI16* locus, as demonstrated by *in vivo* reconstitution experiments in mouse models. While early PMDs, like the *CD1A-IFI16* locus, may emerge solely from selection pressures favoring proliferating prostate cells that escape immune surveillance, it is also possible that such loci have intrinsic properties favoring early loss of DNA methylation.

### Early hypomethylation of core PMDs

The model that hypomethylation-associated gene silencing occurs early and favors tumorigenesis differs conceptually from a hypothesis proposed from a study of advanced colon cancers, whereby hypomethylation might serve an intrinsic tumor suppressor mechanism, restraining uncontrolled cell proliferation^13^. Of note, the colon cancer study analyzed bulk tumor material, encompassing cancer cells together with reactive stroma and immune cells, and it therefore excluded from analysis immune-related genes, whose cell-of-origin is confounded by whole-tumor sequencing. Single-cell level analysis thus allows assignment of all changes in DNA methylation to the appropriate cell type. Most important, however, is our definition of a small subset of PMDs, annotated as core PMDs (2.7% of all PMDs), that appear early in tumorigenesis and are shared uniformly across multiple independent tumors. The identification of early cancer drivers targeted by epigenetic silencing is likely to differ from the contribution of additional PMD-encoded genes that are silenced during subsequent cancer progression, as DNA hypomethylation extends across major portions of the genome. Compared with the small number of core PMDs identified in early cancers, the very large fraction of the cancer genome that is hypomethylated in advanced tumors may thus reflect distinct selection pressures, as well as bystander effects affecting gene-poor PMDs and the derepression of repetitive elements. While our study was centered on prostate cancer, the relevance of core PMDs extends to other cancers, as illustrated by TCGA analyses showing their consistent early hypomethylation across multiple tumors, in contrast to most PMDs which show considerable inter-tumor heterogeneity. Indeed, TCGA methylation data shows that the *CD1A-IFI16* locus to have the strongest difference in DNA methylation between 33 different cancers and their normal tissue counterparts. This specific locus, encoding immune-related genes that have not been previously nominated as critical cancer genes, thus appears to be a consistent target of epigenetic silencing in the early stages of tumorigenesis. Our functional assays using the demethylating agent 5-azacytidine and the EZH2 inhibitor GSK126 support the recruitment of chromatin silencing marks to hypomethylated PMDs as a mechanism of transcriptional silencing. However, further studies will be required to better understand the selectivity of PMD hypomethylation across the genome, and both genomic structure and selection pressures that distinguish core PMDs from more global demethylation.

### The CD1A-IFI16 immune gene cluster

The *CD1A-IFI16* locus is unique in encompassing the entire gene family of *CD1* genes, which together mediate lipid antigen presentation, together with the *IFI16* class of interferon-inducible genes. It is well established that genes that are co-located within a single genomic locus may be targeted during tumorigenesis by either chromosomal deletions or amplification events, a single genetic event that may mediate simultaneous loss-of-function or gain-of-function among physically clustered genes. Conceptually, the hypomethylation silencing of the *CD1A-IFI16* locus during early prostate tumorigenesis may accomplish a similar function, suppressing T cell recognition of lipid antigens as well as double stranded DNA sensing, as part of a single epigenetic event affecting both alleles. Such a potent selective pressure could explain the early and frequent targeting of this locus in cancer. The 1q23.1 genomic locus has been linked in germline association studies to neurodegenerative disease and autoimmune diseases^57,58^, and immunological pathways regulated by its resident genes have been linked to innate immunity against infectious pathogens. The potential roles of these genes in immune surveillance of early cancers will require further functional analyses. Alterations in antigen presentation pathways constitute the most critical mechanisms by which tumors evade both innate and therapeutic immune activation^59,60^. In this respect, the presentation of lipid antigens to NKT cells, a highly specialized subpopulation of T cells, is of particular interest, given potential therapeutic implications. Within prostate cancer, the silencing of *CD1A-IFI16* genes is also noteworthy in that it points to tumor cell-intrinsic factors contributing to the escape from immune surveillance, in addition to the proposed immunosuppressive effects of the tumor microenvironment.

### Diagnostic implications

Finally, from a cancer diagnostic standpoint, blood-based detection of early invasive cancers remains a major technological challenge. For prostate cancer, it requires the ability to distinguish between indolent lesions associated with non-specific elevations in serum PSA and more aggressive cancers that may have similar serum PSA levels but warrant therapeutic intervention. Early invasive prostate cancers shed CTCs into the circulation long before metastases are established^51–53^, and while these rare early CTCs may not be sufficient to cause dissemination, they can serve as potential biomarkers of invasive disease. Microscopic imaging of very rare CTCs in the bloodstream is challenging, hence there is a need for sensitive and quantitative molecular readouts applied to CTC-enriched blood specimens. While this study was not designed to formally test Nanopore sequencing of PMDs as a quantitative molecular surrogate of CTCs, it suggests that such long-range DNA sequencing strategies may complement current approaches that rely on hypermethylation of CpG islands within short ctDNA fragments. Such approaches may also enhance tissue-of-origin determinations, given the information content inherent in such long-range genomic analyses.

### Limitations of the study

Our study suggests that early hypomethylation of core PMDs in prostate cancer differentially silences immune surveillance-associated genes, while sparing genes that mediate cell proliferation. While we find shared patterns of core PMDs across multiple different cancers, it is also possible that distinct tumor types will target alternative biologically relevant pathways. Additional studies in different early stage cancers will be required to distinguish shared hypomethylation targets from those showing tissue-specific patterns, and additional patient-derived samples will need to be analyzed within each tumor type. The potential roles in immune surveillance of lipid antigen presentation genes and IFI16-related double stranded DNA sensing genes deserves further functional analyses using additional experimental systems to define their relevance in early tumorigenesis, as well as their potential relevance for anti-cancer therapy. Finally, the potential utility of PMD detection in blood-based cancer diagnostics will require further validation in larger numbers of diverse clinical specimens.

## STAR Methods

### Resource availability

#### Lead Contact

Further information required to reanalyze the data reported in this paper and requests for resources and reagents should be directed to and will be fulfilled by the lead contact, Daniel A. Haber (dhaber@mgh.harvard.edu).

#### Material Availability

Plasmids generated in this study are available upon written request.

#### Data and Code availability

- All raw and processed sequencing data in this study, including single-cell DNA methylation sequencing, single-cell RNA-seq, ChIP-seq, Cut and Run assay and Nanopore sequencing, have been deposited to the NCBI Gene Expression Omnibus (GEO) database under accession GSE208449. All data are publicly available as of the date of publication.
- This paper analyses existing, publicly available data or available upon request to the authors. These accession numbers for the datasets are listed in the key resources table.
- This paper does not report original code. All the scripts and mathematical algorithms used in this study will be available from the corresponding authors upon request.
- All the versions of software packages used in this study are listed in the key resource table and noted in the data analysis method accordingly.
- Any additional information required to reanalyze the data reported in this paper is available from the lead contact upon request.

### Experimental model

#### Clinical Specimens

All patient samples were collected in this study after written informed consent, in accordance with Institutional Review Board (IRB) protocols (DF/HCC 05–300, 11–497, 13–217 or 14–375). For the CTC cohort, 10–20 ml of blood was drawn from patients with a diagnosis of metastatic prostate cancer, localized prostate cancer, or age-matched males without a diagnosis of cancer at Massachusetts General hospital (MGH). For the localized tumor tissue cohort, all samples were acquired from either core biopsies or surgical resection of untreated localized prostatic adenocarcinoma (Gleason scores 6 and 8) from patients at MGH. In cases with the lowest grade tumors (Gleason score 6), normal prostate tissue was also identified in the tissue specimen by a Genito-Urinary (GU) specialized pathologist and used as a source of matched normal prostate cells. Both normal and tumor tissue samples were de-identified, snap frozen and sectioned. Only tumor sections with >80% tumor content, as assessed by a specialized GU pathologist were used in this study. The clinical data of the patients with metastatic prostate cancer enrolled in the single-cell CTC analysis and patients with resected localized prostate cancer used for single nucleus analysis are described in Table S1. The clinical data of the patients with localized prostate cancer and metastatic prostate cancer enrolled in Nanopore sequencing anlysis of CTC-enriched blood are described respectively in Table S4 and Table S5.

#### Cell culture

Human prostate cancer cell lines (LNCaP, VCaP, PC3 and 22Rv1), murine prostate cancer line (Myc-CaP), normal cultured prostate epithelial cells (HPrEC), benign prostatic hypertrophy cells (BPH-1) and murine Lewis lung carcinoma cells (LLC-1) were all obtained from ATCC, after authentication by short tandem repeat (STR) profiling. All cell lines used in the paper were derived from male mice or male human patients. They were cultured in the following media at 37**°**: RPMI-1640 (ATCC) medium supplemented with 10% FBS (Gibco) and 1X Pen/Strep (Gibco) (for LNCaP, VCaP, PC3, 22Rv1 and BPH-1 cells); Prostate Epithelial Cell medium (ATCC) with 6 nM L-glutamine (ATCC), 0.4% Extract P (ATCC), 1.0 mM Epinephrine (ATCC), 0.5 ng/ml rh-TGFα (ATCC), 100ng/ml hydrocortisone hemisuccinate (ATCC), 5 mg/ml rh-Insulin (ATCC), 5 mg/ml Apo-transferrin (ATCC), 33 μM Phenol red (ATCC) and 1X Pen/Strep/Ampho Solution (ATCC) (for HPrEC cells); DMEM high glucose medium (Gibco) with 10% FBS (Gibco) and 1X Pen/Strep (Gibco) (for Myc-CaP cells and LLC-1 cells). All the cell lines used in this study were checked for mycoplasma every 4 months using Mycoalert kit (Lonza).

#### Mouse xenograft assays

All animal experiments were carried out in accordance with approved protocols by the MGH Subcommittee on Research Animal Care (IACUC). All the mice used in this study were maintained under a 12/12 h light/dark cycle in MGH animal facility. 6–8 weeks old FVB male mice (Jackson Laboratory, Strain#001800) or 6–8 weeks old male immunodeficient NSG (NOD.Cg-Prkdcscid Il2rgtm1Wjl/SzJ) mice (Jackson Laboratory, Strain#005557) were used for intraprostatic injection or subcutaneous injection of Myc-CaP cells stably expressing luciferase and mCherry. 6–8 weeks old C57BL/6 female mice (Jackson Laboratory, Strain#000664) were used for subcutaneous injection of LLC-1 cells stably expressing luciferase. Littermates of the same sex were randomly assigned to experimental groups. For intraprostatic inoculation, mice were first anesthetized using isoflurane, and a 1 cm skin incision was performed along the midline of the abdomen to expose the inner muscle layer, which was then separated. The tip of seminal vesicle was raised gently with forceps to expose the anterior lobe of the prostate gland. 50,000 Myc-CaP cells 1:1 mixed with Matrigel (v/v) (total volume: 30 μl) were slowly injected into the prostate lobe. All the tissues were then returned into the abdomen, and continuous sutures were used to close the inner muscle layer, followed by separate skin closure. For subcutaneous injections, mice were anesthetized, and 50,000 Myc-CaP cells or 1,000,000 LLC-1 cells 1:1 mixed with Matrigel (v/v) (total volume: 100 μl) were injected into the flank. Tumor cell-derived bioluminescent signal was quantified every other day for the Myc-CaP cells and 3 times a week for the LLC-1 for mice after either orthotopic injection or subcutaneous injection. At 2–3 weeks after inoculation, mice were sacrificed and tumors were harvested for flow cytometry and RNA extraction for the Myc-CaP experiments.

### Method Details

#### CTC isolation

CTCs were isolated from fresh blood specimens drawn from patients with prostate cancer, following negative depletion of leukocytes using the microfluidic CTC-iChip as reported previously^26,27^. Briefly, 10–20 ml of whole blood specimens were incubated with biotinylated antibody cocktails against CD45 (R&D Systems, clone 2D1), CD66b (AbD Serotec, clone 80H3), and CD16 (BD Biosciences), followed by incubation with Dynabeads MyOne Streptavidin T1 (Invitrogen) for magnetic labeling and depletion of leukocytes. After CTC-iChip processing, the CTC-enriched product was further stained with FITC-conjugated antibody against EpCAM (Cell Signaling Technology, clone VU1D9) and PE-conjugated antibody against CD45 (BD Biosciences, clone HI30). Single CTCs (FITC positive and PE negative) or white blood cells (WBCs, FITC negative and PE positive) were individually picked into PCR tubes containing 5 μl RNA/DNA lysis buffer using micromanipulator (Eppendorf TransferMan NK 2) and snap-frozen in liquid nitrogen. In total, 38 CTCs from 5 different patients (GU114, GU169, GU181, GU216 and GURa15) with metastatic prostate cancer were individually picked, sequenced and lineage-confirmed based on transcriptome and DNA copy number. One patient sample (GU169) had only one CTC, and it was therefore excluded from some downstream analyses focused on the four patients with multiple CTCs.

#### Nuclei isolation from frozen tumor sections

Tumor tissue sections with high tumor content (>80%) and adjacent normal tissue section were micro-dissected and transferred into a pre-chilled Dounce homogenizer containing ice-cold 1 ml 1X HB buffer (0.26 M sucrose, 30 mM KCl, 10 mM MgCl2, 20 mM Tricine-KOH, 1 mM DTT, 0.5 mM Spermidine, 0.15 mM Spermine, 0.3% NP-40 and 1X complete protease inhibitor). Tissue was homogenized with ~10 strokes of “A” loose pestle, followed by another ~10 strokes of “B” tight pestle. The tissue homogenate was then filtered using a 70 μm strainer and pelleted by centrifugation. Nuclear pellets were resuspended and purified by density gradient centrifugation (top layer: 25% Iodixanol solution; middle layer: 30% Iodixanol solution; bottom layer: 40% Iodixanol solution). The nuclear band at the interface of 30% and 40% Iodixanol solutions was collected into a new Eppendorf tube and washed twice with ice-cold 1X PBS. 20% of the purified nuclei were used to isolate single nuclei using fluorescence-activated cell sorting (FACS) for single-cell DNA methylation analysis, while the remaining 80% of the nuclei were subjected to ChIP-seq analysis.

#### Western Blot

Cells or tumor tissues were lysed in Laemmli buffer (Sigma) and cleared. Protein concentration was determined using DC protein assay (Bio-rad). Proteins (25 μg) were separated on precast NuPAGE 4–12% Bis-Tris protein gels (ThermoFisher), and transferred onto nitrocellulose membranes (Bio-Rad). After blocking with 5% BSA buffer for 1 hour at room temperature, membranes were incubated with primary antibodies overnight at the recommended concentrations. HRP conjugated secondary antibodies (1:10,000; Bio-rad; Cat#5196–2504) were applied, and ultra-sensitive autoradiography film (Amersham) was used to detect the chemiluminescence signal. Primary antibodies used are H3K27me3 (1:1,000, Invitrogen Cat#MA5–11198) and H3 total (1:1,000, Abcam Cat#1791).

#### 5-Azacytidine treatment, bisulfite sequencing and staining of chromatin marks

The human prostate epithelial cell line BPH-1 was cultured in the presence of 5 μM 5-azacitidine (Selleck, #S1782). At serial time points (days 0, 1, 4 and 5), cells were collected for DNA extraction, confocal microscopy, or flow cytometric analysis. DMSO-treated cells were used as control at each time point. To quantify 5-azacitidine-induced demethylation at the genomewide level, we used the whole genome bisulfite sequencing (WGBS). Briefly, DNA ws extracted from BPH-1 cells upon 5-azacitidine treatment, 1 μg genomic DNA was used to sonicate into 300–500 bp fragments, DNA was end-polished, A-tailed and ligated with pre-methylated adaters before bisulfite conversion using EZ DNA methylation kit (Zymo, #D5001), bisulfite-converted DNA was amplified and sample index was introduced during amplification. To quantify 5-azacytidine-induced demethylation at the *CD1A-IFI16* locus, DNA extracted from BPH-1 cells treated with 5-azacitidine was subjected to bisulfite conversion using EZ DNA methylation kit (Zymo, #D5001), and bisulfite-converted DNA was used for PCR amplification, applying bisulfite-specific PCR primers covering the human *CD1A-IFI16* locus (see Table S3). PCR products were purified by 1% agarose gel and cloned using the Zero blunt PCR cloning kit (ThermoFisher, #K270020). 10 individual bacterial clones were randomly picked for Sanger sequencing. Sequencing data were analyzed and shown using online tool QUMA (http://quma.cdb.riken.jp/)^61^. Nuclear accumulation of H3K27me3 was stained with H3K27me3 antibody (1:1000 dilution; CST#9733), in 5-azacytidine-treated cells. Images were acquired using a Zeiss LSM710 Lase Scanning Confocal and were quantified by quantitative image analysis of cells (ImageJ). Flow cytometry was also performed at serial time points on BD LSRFortessa machine to assess CD1d expression using human CD1d-APC antibody (1:100 dilution; BioLegend#350308, clone: 51.1).

#### EZH2 inhibitor treatment

Human prostate cancer cell lines (22Rv1, LNCaP and VCaP) were cultured in the presence of the small molecule EZH2 inhibitor GSK126 (Selleckchem, #S7061) at the indicated concentration (0, 5 or 10μM). After 6 days of treatment, protein and RNA were harvested, for quantitation of H3K27me3 and total H3, using Western blotting and expression of individual genes within the *CD1A-IFI16* locus by real time qPCR.

#### Paired single-cell DNA methylation and RNA-seq

For these experiments, we used either single CTCs or WBCs individually picked from fresh blood specimens after CTC enrichment, and single cells from cultured prostate cell lines (either picked or FACS-sorted). These were subjected to paired single-cell DNA methylation and RNA-seq analysis to obtain the transcriptomes and DNA methylomes from the same single cells^33,62^. Briefly, single cells were first lysed in 5 μl DNA/RNA lysis buffer; 0.5 μl Magnetic MyOne Carboxylic Acid Beads (Invitrogen, Cat#65011) were then added to each single cell lysate to facilitate segregation of nucleus *versus* cytoplasm. After centrifugation and magnetic separation, the supernatant (containing cytoplasmic RNA) was transferred into a new tube for single-cell RNA-seq amplification using the SMART-seq2 protocol^63^, while the pellet (aggregated beads with the intact nucleus) was resuspended in DNA methylation lysis buffer and subjected to single-cell whole genome methylation sequencing using the scBS-seq protocol^64^. Single nuclei sorted from the frozen primary prostate tumor sections were also subjected to the scBS-seq procedure.

#### MNase native ChIP-seq

Purified nuclei from frozen tissue sections were subjected to MNase native ChIP-seq following the ULI NChIP procedure, as published elsewhere^65^. Briefly, nuclei were suspended in Nuclear Isolation Buffer (Sigma) supplemented with 1% TritonX 100, 1% Deoxycholate and 1X complete protease inhibitor. Chromatin was digested by MNase enzyme (NEB, 1:10 diluted) at 21°C for 7.5 min, and further diluted in Complete Immunoprecipitation Buffer, with 1X complete protease inhibitor. 2 μl ChIP-grade H3K27me3 (Active motif, Cat#39155) or H3K9me3 (Abcam, Cat#ab8898) antibody was incubated with the digested chromatin overnight at 4°C. DNA was then purified using protease K digestion followed by phenol-chloroform extraction. ChIP-seq sequencing libraries were prepared using NEBNext Ultra II DNA Library Prep Kit (NEB, Cat#E7645L).

#### Cut and Run Assay

H3K27me3 and H3K9me3 Cut and Run assays were performed with cultured prostate cell lines (LNCaP, 22Rv1, BPH-1, HPrEC and Myc-CaP), using the CUT&RUN Assay kit (CST, Cat#86652S). Briefly, 100,000 freshly cultured prostate cells were collected and incubated with Concanavalin A Magnetic Beads. 2 μl ChIP-grade H3K27me3 (Active motif, Cat#39155) or H3K9me3 (Abcam, Cat#ab8898) or IgG (CST, Cat#66362S) antibody was added to the cell: bead suspension and incubated overnight at 4°C. 1.5 μl pAG-MNase enzyme was then added to the tube, which was rotated for 1 h at 4°C, followed by activation of pAG-MNase using 3 μl cold Calcium Chloride. The activation reaction was stopped and DNA was further diluted and collected for phenol-chloroform extraction. Cut and Run sequencing libraries were constructed using NEBNext Ultra II DNA Library Prep Kit (NEB, Cat#E7645L).

#### Next generation sequencing

All the single-cell RNA-seq, single-cell DNA methylation, MNase ChIP-seq, Cut and Run samples and WGBS samples were molecularly barcoded, pooled together and sequenced on a HiSeq X sequencer to obtain 150 bp pair-ended reads (Novogene).

#### RNA extraction, reverse transcription and quantitative PCR (qPCR)

RNA extracted from cultured prostate cells was prepared using the RNeasy Mini kit (QIAGEN) with DNase I digestion on the column. To extract RNA from mouse tumor tissues, these were first dissected to remove connective tissue and fat, and washed extensively with 1X PBS to remove excessive blood or necrotic tissues. Tumors were then homogenized in RLT RNA lysis buffer using a Dounce homogenizer, and passed through a QIAshredder column (QIAGEN). RNA from normal prostate of FVB mice were prepared following a similar method. RNA from tissue homogenate was extracted using the RNeasy Mini kit (QIAGEN) with DNase I digestion on the column. cDNA was synthesized from 50–200 ng RNA using SuperScript III One-Step qRT-PCR kit (Invitrogen). qPCR was performed using the primers listed in Table S3.

#### CD1d expression measurement by flow cytometry

Cell surface protein expression of CD1d in human and mouse prostate cells was assessed by flow cytometry. Cells were first trypsinized, and 500,000 cells were used for staining with antibody against CD1d at 4°C for 20 min, followed by washing and quantitation using a BD LSRFortessa machine, and data were analyzed using FlowJo software (v10.4; https://www.flowjo.com/). Antibodies used were as follows: for human prostate cell lines, APC conjugated anti-human CD1d (BD#563505, clone: CD1d42) and APC-conjugated isotype control (BD#555751); for Myc-CaP cells, anti-mouse CD1d (Bio X Cell #BE0179, clone 20H2) and the isotype control (Bio X Cell #BE0088), and secondary antibody anti-rat IgG conjugated with APC (Invitrogen #A10540).

#### Plasmid construction

A lentiviral murine *Cd1d1* expression construct (pLenti-Cd1d1-mGFP, Cat#MR226027L4) and its matched control construct (pLenti-C-mGFP, Cat#PS100093) were obtained from Origene. Murine *Ifi20*4 expression vector (pLenti-*Ifi204*-Myc-DDK-Puro, Cat#MR222527L3), together with its control vector (pLenti-C-Myc-DDK-Puro, Cat#PS100092) were also purchased from Origene, and the puromycin selection cassette of these two Origene plasmids were replaced by blasticidin from lentiCRISPRv2-blast plasmid (Addgene#98293) using NEBuilder HiFi DNA Assembly Cloning kit (NEB, Cat#E5520S). For the LLC-1 experiment, the murine *Cd1d1* was cloned into the receiving vector N174-MCS (Addgene#81061) with the restriction enzymes EcoR1 and Mlu1, using the FastDigest protocol of Thermo Scientific. All final construct sequences were confirmed by Sanger sequencing. Plasmids generated in this study are available upon written request.

#### Lentiviral transduction

Early passage 293T cells were transfected with *Cd1d1* or *Ifi204* lentiviral constructs, together with pMD2.G (Addgene#12259) and psPAX2 (Addgene#12260) packaging plasmids using Lipofectamine 2000 reagent (Invitrogen). 48–72 h after transfection, culture medium (containing lentiviral particles) was collected, filtered and concentrated using LentiX concentrator (Clontech). Concentrated virus was added to the Myc-CaP cells in presence of polybrene (Santa Cruz, 8 μg/ml as final concentration) overnight. FACS was used to select GFP positive cells as marker of *Cd1d1* construct transduction in the Myc-CaP cells. The LLC-1 cells transduced with the Cd1d1 cloned in the the N174-MCS vector were selected using G418 (Sigma Aldrich #G8168) at 400 μg/mL for 4–6 days. To obtain stable *Ifi204* overexpression, 10 μg/ml blasticidin (InvivoGen) was added to the medium for 5–7 days selection.

#### Tumor immune infiltration assayed by flow cytometry

Mouse tumors generated by intraprostatic injection of control or *Cd1d1*-expressing Myc-CaP cells were dissected and washed to remove blood, fat and connective tissues. Tumor tissues were further mashed and digested in 5 ml digestion buffer (RPMI1640, 2.5 mg/ml collagenase D, 0.1 mg/ml DNase I) at 37°C for 30 min. Tissue digestion was stopped by adding another 5 ml RPMI1640 with 2% FBS, and then filtered through 70 μm strainers. The tissue cell suspension was obtained in the same way for tumors generated by subcutaneous injection of control or *Ifi204* expressing Myc-CaP cells. To stain for NKT cell infiltration in prostate tumors with control or *Cd1d1* expression, the single-cell suspension was first blocked with rat anti-mouse CD16/CD32 blocking reagent (BD#553142, Clone: 2.4G2) at 4°C for 30 min, followed by mouse NKT surface antibody cocktail staining at 4°C for another 30 min. The mouse NKT surface antibodies used in this study were: BV510-viability dye (BD#564406), APC-α-GalCer-mCD1d Tetramer (TetramerShop#MCD1d-001), BV711-CD69 (BioLegend#104537, clone: H1.2F3), PerCP-Cy5.5-TCRβ (BioLegend#109228, clone: H57–597), BV605-CD3e (BioLegend#100351, clone: 145–2C11) and BUV395-NK1.1 (BD#564144, clone: PK136). Cells obtained from mouse tumors with control or *Ifi204* expression were split into two fractions, with the first fraction stained using a panel of mouse T cell surface antibody cocktails: BV510-viability dye (BD#564406), PerCP-Cy5.5-TCRβ (Biolegend#109228, clone: H57–597), BV711-CD8 (Biolegend#100759, clone: 53–6.7), BV650-CD4 (Biolegend#100546, clone: RM4–5), FITC-CD44 (Biolegend#103006, clone: IM7), PE-Cy7-PD-1 (Biolegend#109110, clone: RMP1–30), BV421-TIM3 (BD#747626, clone: 5D12), APC-TIGIT (Biolegend#156106, clone: 4D4/mTIGIT) and BV785-LAG3 (Biolegend#125219, clone:C9B7W). The second fraction was used to stain for surface and intracellular cytokines by first activating cells with Cell Stimulation Cocktail (eBioscience#00–4970-93) together with Protein Transport Inhibitor Cocktail (eBioscience#00–4980) in 37°C cell culture incubator for 4 h. The cells were then stained for surface antigens before fixation, and subsequently processed for intracellular cytokine staining using BD Fixation/Permeabilization Solution Kit (BD#554714). Antibody cocktails used for surface and intracellular cytokine staining were: BV510-viability dye (BD#564406), PerCP-Cy5.5-TCRβ (Biolegend#109228, clone: H57–597), FITC-CD44 (Biolegend#103006, clone: IM7), PE-TNFα (Biolegend#506306, clone: MP6-XT22), BV650-CD4 (Biolegend#100546, clone: RM4–5), BV711-CD8 (Biolegend#100759, clone: 53–6.7) and BV605-IFNγ (Biolegend#505840, clone: XMG1.2). All flow cytometry was done on the BD LSRFortessa machine, and data were analyzed using FlowJo software (v10.4; https://www.flowjo.com/).

#### Multiplex Oxford Nanopore native sequencing

Blood samples from either healthy donors or patients with localized or metastatic prostate cancer were subjected to CTC-ichip enrichment (10^4^-fold leukocyte depletion)^26,27^. The enriched CTCs (ranging from 0.1% to 1% purity, admixed with residual leukocytes) were subjected to high molecule weight (HMW) DNA extraction using the HMW DNA extraction kit (QIAGEN), and then prepared for Oxford Nanopore sequencing using the rapid barcoding kit (Nanopore#SQK-RBK004). For each sequencing run, 11 blood samples (either from healthy donors or cancer patients), together with 1 lambda DNA (unmethylated control), were uniquely barcoded and pooled together. Sequencing was performed using a Nanopore MinION device with R9.4 flowcell for 48 h, per manufacturer instructions.

#### Single-cell and bulk RNA-seq data analysis

Raw fastq reads generated from HiSeq X sequencer were first cleaned using TrimGalore (v0.4.3) (https://github.com/FelixKrueger/TrimGalore) to remove the adapter-polluted reads and reads with low sequencing quality. Cleaned reads were aligned to the human (hg19) or mouse (mm9) genome using Tophat (v2.1.1)^66^. PCR duplicates were further removed using samtools (v1.3.1)^67^, gene counts were computed using HTseq (v0.6.1)^68^, gene expression level (FPKM) was further calculated using cufflinks (v2.1.1)^66^. Gene expression matrix was subjected to R (v3.1.2) or Prism9 for graphics.

#### Single-cell and bulk DNA methylation sequencing data analysis

Raw fastq reads from both the single-cell and bulk DNA methylation sequencing were first trimmed using TrimGalore (v0.4.3) (https://github.com/FelixKrueger/TrimGalore), and cleaned reads were aligned to the human hg19 or mouse mm9 genome (in silico bisulfite converted) using Bismark tool (v0.17.0)^69^. Samtools (v1.3.1)^67^ was used to remove PCR duplicates, and CpG methylation calls were extracted using the Bismark methylation extractor^69^. 0.1% lambda DNA was spiked in, prior to bisulfite treatment, for each sample to assess the bisulfite conversion efficiency. Only samples with more than 4 million unique CpG sites covered at least once and with a bisulfite conversion rate > 98% were used in this study.

#### TCGA methylation array data reanalysis

Prostate DNA methylation datasets from TCGA analyzed by Illumina Infinium Human Methylation 450 K BeadChip were downloaded from the National Cancer Institute’s GDC Data Portal (https://portal.gdc.cancer.gov) for 502 tumor samples and 50 normal samples. CpG site-level methylation files (beta value, txt format) were first converted to hg19 coordinates using UCSC lift-over tool (https://genome.ucsc.edu/cgi-bin/hgLiftOver) for the downstream analysis. The data were binned to a fixed set of 10 kb nonoverlapping genomic windows by computing the average fraction methylation within each bin in each sample. Bins were excluded if they lacked coverage (i.e., had no probes on the Illumina Infinium Human Methylation 450 K BeadChip array) or had a mean normal-tissue methylation level, averaged across all the normal samples, of <70%. For each sample, the global methylation level was calculated as the fraction of bins having methylation >50%. The methylation level at the *CD1A-IFI16* locus for each sample was calculated as the fraction of bins in the range chr1:158,130,000–158,340,000 (hg19) having methylation >50%. The gene expression data and clinical information of TCGA PRAD samples, including Gleason score, tumor stage and others, were all downloaded from cbioportal (https://www.cbioportal.org/). Tumor purity was calculated using ABSOLUTE algorithm^70^. DNA Methylation 450 K BeadChip datasets for other cancer types were also downloaded from the National Cancer Institute’s GDC Data Portal (https://portal.gdc.cancer.gov) and CpG site-level methylation files (beta value, txt format) were also converted to hg19 coordinates using UCSC lift-over tool (https://genome.ucsc.edu/cgi-bin/hgLiftOver) for the downstream analysis.

#### Genomic element enrichment analysis

For analytical purposes, a promoter region was defined based on the relative position to a transcription start site (TSS): 1,500 bp upstream and 500 bp downstream. The annotations of TSS, exon, intron, intragenic regions, CpG islands (CGIs), repetitive elements and UCSC gap regions were all downloaded from UCSC genome table browser (https://genome.ucsc.edu/cgi-bin/hgTables)^71^. Enrichment analysis on different genomic elements was calculated using the Bioconductor package regioneR (v1.18.1) with overlapPermTest function^72^.

#### DNA copy number analysis inferred by single-cell DNA methylation sequencing data

Single-cell DNA methylation sequencing reads were first aligned to the genome using Bismark. Uniquely aligned reads were extracted into a bed file and subsequently submitted to Ginkgo online tool^73^, http://qb.cshl.edu/ginkgo) to infer the DNA copy number, using 5 Mb as the bin size. The processed integer copy number data from the Ginkgo website (SegCopy.tsv) was used to calculate the DNA Copy Number Variation (CNV) score. Given an assignment of a copy number to all the locations in a diploid genome, we define a CNV score for any given single cells as follows. Let c*i* be the copy number at the *i*th location of the genome. CNV score is then defined to be the average over all *i* in the genome of the absolute value of (c*i*-2).

#### DNA copy number analysis inferred by single-cell RNA-seq data

Single-cell RNA-seq reads were aligned to human genome using TopHat, and large-scale chromosomal copy number alterations were determined by InferCNV (https://github.com/broadinstitute/infercnv).

#### MNase ChIP-seq and Cut and Run data analysis

ChIP-seq and Cut and Run reads were first trimmed by Trim Galore (v0.4.3) (https://github.com/FelixKrueger/TrimGalore) and then mapped to the human or mouse genome using BWA men^74^. Duplicated reads were marked by sambamba^75^ and further removed using samtools^67^. MACS2 (v2.0.10)^76^ was used to call the peaks and deepTools^77^ were used to compute the ChIP-seq or Cut and Run signal around prostate PMDs.

#### Determination of Partially Methylated Domains (PMDs)

The human genome was first binned into 100 kb windows placed at 200 bp offsets. Windows that intersected CGIs or UCSC gap regions were discarded. For each source (i.e., single CTCs from patients with prostate cancer, single WBCs from healthy donors, single cells from normal prostate or prostate cancer cell lines or normal prostate tissues^42^, the per-source methylation level of each window was calculated by taking the average over all cells from that source of the methylation level of the CpG sites within the given window. For each source the distribution of the per-source methylation level of the 100 kb windows was plotted. Normal cells showed a unimodal distribution, while prostate cancer cells showed a bimodal distribution. A threshold for hypomethylation determination was set at the lowest point of the valley in the histogram of the bimodal distribution for each prostate cancer patient or prostate cell line; if the distribution was unimodal, the threshold was set to 60%. The windows with methylation level lower than threshold were defined as hypomethylation windows and overlapping hypomethylation windows were merged into per-source PMDs. The 250 kb minimal length threshold was then applied to the per-source PMDs. The *union* of the per-source PMDs for all single CTCs from four prostate cancer patients (GU114, GU216, GURa15 and GU181) and for all single cells from four prostate cancer cell lines (LNCaP, VCaP, 22Rv1 and PC3) was defined as the total prostate PMDs (1,496 in total). Chromatin mark and genome element enrichment analyses were performed on these PMDs. To identify the genes that reside in the most consistently hypomethylated PMDs across all prostate cancer specimens analyzed (i.e., *intersection*), we quantile-normalized the DNA methylation levels for all PMDs among all CTCs from four prostate cancer patients (GU114, GU216, GURa15 and GU181) and all single cells from four prostate cancer cell lines (LNCaP, VCaP, 22Rv1 and PC3) and only used the PMDs (annotated as core prostate PMDs) with their averaged quantile-normalized DNA methylation level less than 25% across these 8 sources to extract the genes.

#### Determination of Preserved Methylation Islands (PMIs)

After identification of PMDs for each of the eight sample sources [CTCs from four prostate cancer patients (GU114, GU216, GURa15 and GU181) and single cells from four prostate cancer cell lines (LNCaP, VCaP, 22Rv1 and PC3)], we defined small interspersed islands (“gaps”) with preserved methylation (sample source PMIs) using the following criteria: (1) every PMI is flanked by defined PMDs in each given source; (2) length of each PMI should be >30 kb and <3 Mb. Total prostate PMIs were defined by taking the *union* of sample source PMIs across 8 sources (1,412 in total), while core prostate PMIs (44 in total) were defined by requiring the uniformity across sample sources: the genomic location of given PMI is overlapped in all 8 sample sources.

#### Differential gene expression and hypergeometric gene set enrichment analysis (hGSEA)

Differential gene expression between TCGA prostate normal tissue and primary tumors was determined as follows: We started by considering the genes that reside in the most hypomethylated PMDs [as described in the section titled “Determination of partially methylated domains (PMDs)”]. Of those, genes with 95th percentile of normalized FPKM values less than 1 were discarded. A two-tailed variance-equal t-test was performed on each of the remaining genes. The p-values from those t-tests were used to generate a false-discovery rate (FDR) estimate for each gene by the Benjamini-Hochberg method. We considered genes for which the FDR estimate was less than 0.1 to be differentially expressed between normal prostate and prostate tumor samples. hGSEA was performed to determine the gene set and pathway enrichment using the phyper R function as reported elsewhere^26^. All gene sets and pathways evaluated in this study were obtained from MSigDB (v7.2) from the Broad Institute. Differential gene expression and hGSEA for genes in PMIs was performed in the same way.

#### Heterogeneity assessment

Consistent with a previous publication^26^, means of correlation coefficients and jackknife estimates were used to assess the heterogeneity within and between subsets of samples.

#### Nanopore data analysis

Nanopore sequencing reads (format: fast5) generated by Nanopore MinION device were first converted into fastq files using ONT Albacore software (v2.3.1) (https://nanoporetech.com/community). Demultiplexing was also performed during fast5 to fastq conversion. DNA methylation information was extracted from both fast5 and fastq files using Nanopolish software (v0.10.2) (https://github.com/nanoporetech/nanopolish). Nanopolish output files (albacore_output.sorted.bam and methylation_calls.tsv) were used for downstream analysis. Every nanopore run was spiked in with lambda DNA, which was used as the negative control to assess the fidelity of Nanopore sequencing. To estimate CTC-derived hypomethylation signal in each Nanopore sequencing sample, stringent criteria were applied: (1) each Nanopore read should be long enough to harbor at least 30 CpG sites with confident methylation calls after Nanopolish; (2) the number of Nanopore reads aligned to prostate PMDs (pre-determined among CTCs isolated from 4 prostate cancer patients and 4 prostate cancer cell lines using single-cell whole genome bisulfite sequencing) should be no fewer than 300 for metastatic patients or no fewer than 400 for localized patients; (3) methylation level of spike-in lambda DNA in each run should be <1%. Following application of these criteria, microfluidic processed (leukocyte-depleted) blood samples from seven patients with metastatic prostate cancer, six patients with localized prostate cancer. Since we required different number of Nanopore reads in the prostate PMDs for metastatic patients and localized patients, 23 age-matched healthy donors were validated for analysis in the metastatic cohort, and 21 were validated for localized cohort.

#### *In-silico* mathematical modeling of Nanopore sequencing in detecting rare signal

To assess the ability to detect large hypomethylated domains in rare circulating tumor cells, we performed an analysis using Nanopore reads from a normally methylated non-cancer cell line (HUES64) with 1% *in-silico* spiked-in reads from a cancer cell line (HCT116). We assessed the ability to determine the correct cell line of origin for reads that aligned to predefined HCT116 PMDs based on their average methylation level by quantifying the precision and sensitivity of read classification using the PRROC^78^. Methylation was averaged across each read, considering only CpG sites that fall within PMDs and excluding those within CpG islands.

#### Illustration

Illustrations were created with BioRender.com.

### Quantification and Statistical Analysis

Statistical analyses for all experiments are described in the figure legends and the method details. Statistical analyses were performed using R (version 3.1.2) and GraphPad Prism 9.0.

## Supplementary Material

1 Table S1 Clinical characteristics of patients with metastatic prostate cancer enrolled in single-cell CTC analysis and patients with resected localized prostate cancer used for single nucleus analysis, related to Figure 1.
Abbreviations: ADT, androgen deprivation therapy; Brachy, brachytherapy; CRPC: castration-resistant prostate cancer; RP, radical prostatectomy; EBRT, external beam radiation therapy; sip-T, sipuleucel-T; PSA, serum prostate-specific antigen.

2 Table S2 Chromosomal locations with respect to hg19 of total PMDs, core PMDs, total PMIs and core PMIs, related to Figures 1 and 2.

3 Table S3 Primer sequences used in this study, related to Figure 5, S6 and S8.

4 Table S4 Clinical characteristics of patients with localized prostate cancer enrolled in Nanopore sequencing analysis of CTC-enriched blood samples, related to Figure 6.
Abbreviations: PSA, serum prostate-specific antigen; N/A, not applicable.

5 Table S5 Clinical characteristics of patients with metastatic prostate cancer enrolled in Nanopore sequencing analysis of CTC-enriched blood samples, related to Figure 6.
Abbreviations: ADT, androgen deprivation therapy; PSA, serum prostate-specific antigen; Ra223, radium-223; RT, radiation therapy; N/A, not applicable.

6 **Figure S1. Single-cell transcriptome and DNA copy number analysis of prostate CTCs, related to** Figure 1.
**(A-B)** Boxplots showing sequencing quality parameters of single-cell DNA methylation samples (panel **A**) and single-cell RNA-seq samples (panel **B**).
**(C)** Heatmap showing marker gene expression of the single cells sequenced in this study. CTCs have high expression of epithelial and prostate lineage markers, and absent expression of leukocyte (WBC) markers. Single WBCs that persisted after processing through the microfluidic device are shown as negative controls, and single cells from prostate cancer cell lines are used as positive controls. Asterisks denote a small number of CTCs with potential contamination by WBCs, which were excluded from analysis.
**(D)** Heatmap showing unsupervised hierarchical clustering of single-cell RNA-seq. Three major clusters are defined (upper dendrogram): WBC; normal prostate cell line cells (HPrEC and BPH-1); and prostate CTCs together with four prostate cancer cell lines (PC3, LNCaP, VCaP and 22Rv1).
**(E-F)** Heatmaps showing matched DNA copy number of CTCs inferred from single-cell DNA methylation sequencing data (panel E) and from single-cell RNA-seq data (panel **F**).

7 **Figure S2. DNA methylation analysis of single prostate CTCs, related to** Figure 1.
**(A)** PCA analysis of promoter methylation in single prostate CTCs, and single cells from prostate cancer cell lines (22Rv1, LNCaP, PC3 and VCaP), non-transformed prostate epithelial cell lines (HPrEC and BPH-1), residual WBCs following microfluidic processing, and normal prostate tissues. All prostate tumor cells cluster separately from both non-transformed prostate cells and from normal leukocytes.
**(B)** Histograms showing distribution of methylation level within each 100 kb window, with coverage of at least 10 CpGs at 200bp offsets across the genome, representing averages from single-cell data for patient-derived CTCs (grouped by patient: GU114, GU181, GU216, GURa15), prostate cancer cell lines (LNCaP, VCaP, PC3 and 22Rv1), normal prostate tissue, non-transformed prostate epithelial cell lines (HPrEC and BPH-1), whole blood (representing all hematopoietic lineages) and leukocytes (WBC). The blue vertical line in each cancer-related specimen is the threshold set to score hypomethylation in that cell type (i.e., every 100 kb window with methylation levels below that threshold is defined as hypomethylated).
**(C)** Bar graph showing the fraction of the genome that is hypomethylated in patient-derived CTCs, prostate cancer cell lines, and normal cell lines or tissues. All tumor samples have 20–40% of their genome classified as hypomethylated, while the normal samples have <2.5%. N.P., normal prostate.
**(D)** Flowchart depicting the key steps of definition of prostate PMDs and PMIs (see Methods).

8 **Figure S3. Chromatin silencing marks and size of core PMDs and core PMIs, related to** Figure 2.
**(A)** Line plots showing differential enrichment for H3K27me3 marks at PMDs in prostate cancer cells (22Rv1, red) compared with non-transformed prostate epithelial cell lines (BPH-1, green and HPrEC, blue) (left panel). In contrast, there is no significant difference in the abundance of H3K9me3 at PMDs between cancer cells and normal cells (right panel).
**(B)** Boxplot quantifying the enrichment for H3K27me3 at all prostate PMDs in 22Rv1 prostate cancer cells, compared with normal prostate HPrEC and BPH-1 cells. No enrichment is observed for H3K9me3. P-value assessed by one-tailed Student’s t test.
**(C)** IGV screenshot (hg19) of the *CD1A-IFI16* locus at chromosome 1, showing DNA hypomethylation (shade yellow) in 22Rv1 prostate cancer cells (red), compared with HPrEC and BPH-1 prostate epithelial cells (blue and green). The *CD1A-IFI16* locus also shows enrichment for H3K27me3 chromatin silencing marks in prostate cancer cells, compared with normal prostate cells, but no such differential abundance for H3K9me3 silencing marks.
**(D)** Inter- and intra-patient heterogeneity analysis of PMIs among prostate CTCs and single cells from prostate cancer cell lines. Mean Jaccard index is used to indicate the heterogeneity, with higher mean Jaccard index score indicating less heterogeneity among samples assayed. Error bar indicates mean with 95% CI.
**(E-F)** IGV representation (hg19) of total PMIs and core PMIs at a chromosome 1 locus, across 8 sample sources (4 prostate patients and 4 prostate cancer cell lines). Total PMIs (blue) are the union of all PMIs defined in each sample source, while core PMIs (black) are those shared across all 8 sample sources (panel **E**); representation of PMIs from the single-cell components of an individual sample source (22 CTCs from patient GU181) showing a core PMI (black) shared across all sample sources and neighboring non-core PMIs (red) that are shared by >85% of CTCs in this patient, but not across different sample sources (panel **F**).
**(G)** Bar graph showing significantly reduced mean CpG density at core PMDs, compared to other PMDs. Blue line indicates the mean CpG density of 40 core PMDs. Histogram represents the mean CpG densities of 10,000 random samplings of 40 non-core PMDs. P-value is the fraction of the random samplings of non-core PMDs for which the mean CpG density is less than the mean CpG density of the 40 core PMDs.
**(H)** Boxplots showing the average length of total PMDs, total PMIs, core PMDs and core PMIs across the prostate cancer genome.

9 **Figure S4. Demethylation of *CD1A-IFI16* locus at early stage of prostate tumorigenesis, related to** Figure 4.
**(A)** Heatmap showing DNA copy number variation (CNVs) within single cells retrieved from adjacent normal tissues, low-grade localized prostate cancer (GS6), high-grade localized prostate cancer (GS8), and metastatic prostate cancer (CTCs). Single-cell DNA methylation sequencing data were used to infer CNVs. Box marked by dashed red line denotes chromosomal deletion of the chr8p locus, which appears as the earliest and most consistently observed CNV in early prostate cancer.
**(B)** Boxplots showing concordance of hypomethylation and chr8p deletion within single cancer cells at the one of the earliest stage of prostate tumorigenesis (GS6), across the genome, at all PMDs, and at the *CD1A-IFI16* locus. The correlation is lost at more advanced stages of prostate cancer (GS8 and CTCs), when CNV and hypomethylation are pronounced and distributed across the genome. P-value, all assessed by Wilcoxon test.
**(C-D)** Heterogeneity of promoter methylation (panel **C**) and PMD methylation (panel **D**) within individual cells from localized prostate cancer (GS6 and GS8), metastatic prostate cancer (CTCs) and prostate cancer cell lines, measured by mean correlation coefficient, and showing the relative uniformity of PMD hypomethylation in prostate cancer, compared with promoter hypermethylation. Error bar denotes mean with 95% CI.
**(E)** Bar plots showing gradual increase of methylation at CpG islands (CGIs) during prostate cancer progression. Error bar denotes mean with SD. P-value was assessed by two-tailed Student’s t test.
**(F)** Quantitation of DNA copy number alterations during prostate tumorigenesis, with normal prostate showing no CNV, and gradual increase in CNV from GS6, to GS8, and CTCs. Error bar indicates geometric mean with 95% CI.
**(G)** Scatter plot showing correlation between DNA copy number alterations and DNA methylation changes in GS6 and GS8 tumors and in prostate CTCs. Each dot indicates one core PMD. X-axis indicates the relationship (rho) between DNA copy number alterations and DNA methylation changes at the corresponding regions (negative correlation at left, and positive correlation at right), y-axis indicates the FDR, with dash line showing FDR of 0.1.
**(H)** Quantitation of progressive demethylation at core PMDs (red) *versus* non-core PMDs (magenta) as a function of evolution from normal prostate tissues (n=4), primary prostate tumors (n=5) (derived from Yu et al., 2013) and metastatic prostate tumors (n=100) (derived from Zhao et al., 2020) showing the more rapid loss of DNA methylation at core PMDs. In contrast, core PMIs (blue), which are interspersed between core PMDs, show preservation of DNA methylation during cancer progression. Methylation level was calculated by reanalyzing the two published datasets. Error bar denotes mean with SEM. Statistical analysis of DNA methylation curves using longitudinal linear mixed effects model, by which tumor progression (normal tissue, primary prostate tumor, and metastatic prostate tumor) x methylation domains was tested.
**(I)** Boxplots showing mean expression level in normal prostate and prostate tumors (TCGA) of E2F target pathways and DNA repair pathways, whose genes reside within core PMIs. P-value assessed by Wilcoxon test.
**(J)** Heatmap representation of genes residing within PMIs, and belonging to the E2F targets and DNA repair pathways, showing preserved expression in primary prostate cancers, compared with normal prostate tissue (TCGA).

10 **Figure S5. DNA methylation analysis at the *CD1A-IFI16* locus in prostate and other cancers, related to** Figure 4.
**(A-C)** DNA methylation changes as a function of Gleason score, in microdissected single nuclei from surgically resected specimens of localized prostate cancer **(A)**, in samples derived from two independent public datasets of prostate tumors (normal prostate tissue and primary prostate tumors derived from Yu et al, 2013, and metastatic prostate tumors derived from Zhao et al, 2020) **(B)** and from Gleason-annotated TCGA prostate tumor specimens **(C)**. Line plots show a marked loss of DNA methylation at the *CD1A-IFI16* locus (green line), in contrast to genomewide DNA methylation (orange line). Of note, **(A)** and **(B)** data derived from WGBS, and **(C)** data from Infinium Human Methylation 450K BeadChip. Error bar denotes mean with SEM. Statistical analysis of DNA methylation curves using longitudinal linear mixed effects model, by which tumor progression (or Gleason Score) x methylation domains was tested.
**(D)** Boxplots showing absence of significant methylation changes across single cells (normal, GS 6, GS 8, CTCs) representing different grades of prostate cancer, as a function of DNA copy number variation at the *CD1A-IFI16* locus. ns, not significant; *P<0.05, assessed by Wilcoxon test.
**(E)** Line graphs showing earlier demethylation at the *CD1A-IFI16* locus, compared with other core PMDs, during colon cancer and thyroid cancer progression. Error bar denotes mean with SD. P-value, assessed by two-tailed Student’s t test.
**(F)** IGV screenshot (hg19) showing DNA methylation at a region of chromosome 1 encompassing the *CD1A-IFI16* locus, across 33 different cancer types (TCGA). 23 (denoted in red) show hypomethylation (<80%) of the *CD1A-IFI16* locus. Abbreviations for cancer types: ACC, Adrenocortical Carcinoma; BLCA, Bladder Urothelial Carcinoma; BRCA, Breast Invasive Carcinoma; CESC, Cervical Squamous Cell Carcinoma and Endocervical Adenocarcinoma; CHOL, Cholangiocarcinoma; COAD, Colon Adenocarcinoma; DLBC, Lymphoid Neoplasm Diffuse Large B; ESCA, Esophageal Carcinoma; GBM, Glioblastoma Multiforme; HNSC, Head Neck Squamous Cell Carcinoma; KICH, Kidney Chromophobe; KIRC, Kidney Renal Clear Cell Carcinoma; KIRP, Kidney Renal Papillary Cell Carcinoma; LAML, Acute Myeloid Leukemia; LGG, Brain Lower Grade Glioma; LIHC, Liver Hepatocellular Carcinoma; LUAD, Lung Adenocarcinoma; LUSC, Lung Squamous Cell Carcinoma; MESO, Mesothelioma; OV, Ovarian Serous Cystadenocarcinoma; PAAD, Pancreatic Adenocarcinoma; PCPG, Pheochromocytoma and Paraganglioma; PRAD, Prostate Adenocarcinoma; READ, Rectum Adenocarcinoma; SARC, Sarcoma; SKCM, Skin Cutaneous Melanoma; STAD, Stomach Adenocarcinoma; TGCT, Testicular Germ Cell Tumors; THCA, Thyroid Carcinoma; THYM, Thymoma; UCEC, Uterine Corpus Endometroid Carcinoma; UCS, Uterine Carcinosarcoma; UVM, Uveal Melanoma.
**(G)** Plot showing methylation levels across 33 different cancer types at the *CD1A-IFI16* locus (right), compared with genome-wide methylation (left). The 23 cancers with hypomethylation (<80%) at the *CD1A-IFI16* locus are colored in red. Abbreviations defined in **(F)**.
**(H)** Scatter plots showing correlation between DNA hypomethylation at the *CD1A-IFI16* locus and reduced expression of resident genes across 33 different cancer types (TCGA). Statistically significant (FDR<0.1) correlations between hypomethylation and reduced RNA expression are shown with green line (19 tumor types); anti-correlations shown with red line (4 tumor types); tumor types which do not reach statistical significance with yellow line (10 tumor types). Abbreviations defined in **(F)**.

11 **Figure S6. Chromatin silencing marks and transcriptional changes at the *CD1A-IFI16 locus,* related to** Figure 4.
**(A)** Boxplots quantifying increased H3K27me3 marks, but unchanged H3K9me3, in prostate cancer cells compared with normal cells, at the *CD1A-IFI16* locus, using Cut and Run assays. Two prostate cancer cell lines (LNCaP and 22Rv1) are compared with the two non-transformed prostate epithelial cell lines (HPrEC and BPH-1). P-value is assessed by one-tailed Student’s t test.
**(B)** Plots showing reduced expression (qRT-PCR) of interferon inducible genes at the *CD1A-IFI16* locus (*IFI16, PYHIN1* and *AIM2*) in two prostate cancer cell lines (LNCaP and 22Rv1), compared with two non-transformed epithelial prostate cell lines (HPrEC and BPH-1). Error bar denotes mean with SD. P-value assessed by two tailed Student’s t test.
**(C)** Flow cytometric quantitative analysis of CD1d expression in two prostate epithelial cell lines (BPH-1 and HPrEC), compared with two prostate cancer cell lines (LNCaP and 22Rv1), showing reduced CD1d expression in the tumor cells. Cells were incubated with the APC conjugated anti-human CD1d or with the APC-conjugated isotype control IgG (shown in grey).
**(D)** IGV screenshot (hg19) showing enrichment for H3K27me3 ChIP-seq signal at the *CD1A-IFI16* locus, as early as GS 6 during early prostate tumorigenesis. H3K27me3 ChIP-seq is shown for two normal prostate tissues (grey tracks), two GS 6 tumor tissues (red tracks) and four GS 8 tumor tissues (green tracks).
**(E-F)** Boxplots showing quantitative enrichment for the chromatin silencing mark H3K27me3 during progression from normal prostate epithelium to stages of localized prostate cancer (GS 6, GS 8). Increased H3K27me3 is evident as early as GS 6 (**panel E**; representative IGV track (hg19) shown in panel D), whereas all PMDs do not show statistically significant increased deposition of H3K27me3 until GS 8 (**panel F**). P-value all assessed by one-tailed Student’s t test.
**(G)** Plots showing reduced expression of all members of the *CD1A-E* lipid antigen presentation gene family and three out of four interferon inducible genes, all colocalized at the *CD1A-IFI16* locus, within primary prostate tumors *versus* normal prostate (TCGA prostate data with tumor purity >0.5 inferred by ABSOLUTE algorithm). Error bar denotes mean with SEM. P-value assessed by two-tailed Student’s t test.

12 **Figure S7. Functional recapitulation of hypomethylation-associated silencing at *CD1A-IFI16* locus, related to** Figure 4.
**(A)** IGV screenshot (hg19) of bulk DNA methylation profile at the *CD1A-IFI16* locus (delineated by hashed red box) showing DNA demethylation in the human prostate epithelial cells BPH-1 following 4–5 days of treatment with 5-azacytidine, compared with DMSO controls and day 1 after treatment.
**(B)** Lollipop graph showing region within the *CD1A-IFI16* locus (12 CpG sites over 395 bp), using bisulfite PCR coupled with Sanger sequencing from BPH-1 cells at serial timepoints after treatment with 5-azacytadine. Methylated CpGs (black circles), unmethylated CpGs (open circles), with mean DNA methylation fraction indicated below each panel.
€ Representative confocal microscopic images of BPH-1 cells showing increased nuclear abundance of the H3K27me3 chromatin silencing mark, following 5 days of treatment with 5-azacytidine (*versus* DMSO control). DNA content is labelled with DAPI (blue). Red fluorescence indicated H3K27me3. One magnified representative nucleus is shown in the lower left corner. Bar 50 μM.
**(D)** Representative confocal microscopic images of BPH-1 cells showing increased nuclear abundance of the H3K9me3 chromatin silencing mark, following 5 days of treatment with 5-azacytidine (*versus* DMSO control). DNA content is labelled with DAPI (blue). Red fluorescence indicated H3K9me3. One magnified representative nucleus is shown in the lower left corner. Bar 50 μ€**(E)** Quantitation of confocal microscopic imaging of mean H3K9me3-related fluorescence intensity within single-cell nuclei (quantitation using ImageJ software, see methods). Error bar denotes mean with SEM. P-value assessed by two tailed Student’s t test.
**(F-G)** Induction of genes residing at the *CD1A-IFI16* locus in three human prostate cancer cell lines (22Rv1, LNCaP and VCaP), which harbor PMD hypomethylation and H3K27me3 deposition, following their treatment of with the EZH2 inhibitor GSK126 for six days (5μM and 10μM doses). GSK126 exposure leads to profound reduction in H3K27 trimethylation (Western blots, panel **F**) along with increased expression of all genes within the *CD1-IFI16* gene cluster (quantitative real-time PCR, panel **G**). No change is observed in the expression of non-PMD resident control genes (*PP1A*, *HPRT* and *β-actin*). P-value assessed by Tukey’s multiple comparison tests, where GSK126 treatment conditions were compared to their control (blue bar). N.s. not significant; *P<0.05**P<0.01; ***P<0.001; ****P<0.0001.

13 **Figure S8. Re-expression of the lipid antigen presentation gene *Cd1d1* or the interferon inducible gene *Ifi204* suppresses tumorigenesis in immune competent murine prostate cancer models, related to** Figure 5.
**(A-B)** IGV screenshots (mm9) showing DNA hypomethylation (shaded yellow) and occupancy of the repressive histone marks H3K27me3 and H3K9me3 in mouse Myc-CaP prostate cancer cells, at the two murine loci that are orthologous to human *CD1A-IFI16: Cd1d1* and *Cd1d2* genes are clustered on mouse chromosome 3 (panel **A**), and interferon-inducible genes *Ifi204, Aim 2, Pyhin1* and *Mnda* are clustered on mouse chromosome 1 (panel **B**). H3K9me3, H3K27me3 and IgG (control) are shown with two biological replicates. DNA methylation derived from single-cell whole genome bisulfite sequencing of 12 single Myc-CaP cells.
**(C)** Flow cytometric analysis of lentivirally-mediated ectopic *Cd1d1* overexpression (OE) in Myc-CaP cells, showing cell surface localization of the encoded protein, compared with empty vector transfected control and IgG staining control.
**(D)** Confocal microscopic image showing cell surface localization of CD1d after ectopic expression in Myc-CaP cells. Green florescence indicates CD1d expression (white arrows), Myc-CaP cells are labeled with mCherry. Bar, 20 μM.
**(E)** *In vivo* intraprostatic orthotopic tumorigenesis assay, showing the relatively rapid clearance of Myc-CaP cancer cells with ectopic *Cd1d1* expression, compared with parental control. Representative mouse images at day 9 are shown. Error bar denotes mean with SEM. P-value, assessed by two-tailed Student’s t test.
**(F)** Plot showing quantitative (qRT-PCR) gene expression difference of NKT cell-specific RNA markers (*Cd40lg* and *Icos*) and pan-leucocyte marker (*Ptprc, CD45*), between tumors generated by Myc-CaP cells with restored *Cd1d1* expression (0E) versus mock-transfected controls. P-value is assessed by two-tailed Student’s t test.
**(G)** qRT-PCR quantitation showing expression of *Cd1d1* expression in LLC-1 cells after lentiviral transduction. The magnitude of differential expression (140-fold) reflects the virtually complete absence of *Cd1d1* RNA expression in parental LLC-1 cells. P-value assessed by Student’s t-test.
**(H)** Flow cytometric quantitative analysis of Cd1d protein expression in LLC-1 cells, following lentivirally-mediated ectopic expression, compared with mock transduced control and IgG staining control. Cell surface expression of Cd1d1 is comparable to that in transduced Myc-CaP cells (panel **C**).
**(I)** *In vitro* cell proliferation assay showing no difference between LLC-1 cells with overexpression (OE) of *Cd1d1*, compared with parental controls. Error bar denotes mean with SD; ns, not significant, assessed by two-tailed Student’s t test.
**(J)** Quantitation of tumor formation in isogenic C57BL/6 mice, following subcutaneous inoculation of luciferase-tagged LLC-1 cells with overexpression of *Cd1d1* or mock-transduced controls, using bioluminescence IVIS imaging. P-value assessed by Student’s t-test. N= 8 in each group.

14 **Figure S9. Flow cytometric analysis of immune infiltration in subcutaneous Myc-CaP-derived tumors, related to** Figure 5.
**(A)** Flow cytometric analysis showing no significant difference in tumor infiltration by CD3e^+^NK1.1^+^ double positive cells (total NKT cells, not CD1d-restricted) in tumors derived in immune competent isogenic mice by Myc-CaP cells with *Cd1d1* overexpression (OE) or mock-transduced controls. Quantitative analysis in right panel. ns, not significant; P-value is assessed by two-tailed Student’s t test.
**(B-C)** Flow cytometric analysis showing enrichment of CD1d-restricted NKT cells (marked by α-GalCer CD1d Tetramer; panel **B**) and activated NKT cells (marked by CD69 expression; panel **C**) in tumors derived from Myc-CaP cells with *Cd1d1* overexpression (OE), compared to mock-transduced controls. Quantitation of positive cells (percent) shown in upper right.
**(D)** Gating strategy for scoring of different cell surface markers of infiltrated T cells in one representative tumor sample.
**(E)** No difference in percent of CD44 expressing CD4^+^ or CD8^+^ T cells in parental control *versus Ifi204* expressing tumors. ns, not significant, assessed by two tailed Student’s t test.
**(F-G)** FACS plots showing downregulation of PD-1 cell surface expression (panel **F**) and upregulation of TNFα (panel **G**) in CD8^+^ T cells recovered from Myc-CaP-derived tumor with overexpression (OE) of *Ifi204*, compared with mock-transduced controls.
**(H-K)** No difference in LAG3 (panel **H**), TIGIT (panel **I**), TIM3 (panel **J**) and IFNγ (panel **K**) expression in CD8^+^ T cells in control *versus Ifi204* expressing tumors. Error bar denotes mean with SD. ns, not significant, assessed by two tailed Student’s t test.

## Figures and Tables

**Table:** Key resources table

REAGENT or RESOURCE	SOURCE	IDENTIFIER
Antibodies		
Mouse anti-CD45 biotinylated (clone 2D1)	R&D Systems	Cat# BAM1430; RRID:AB_356874
Mouse anti-CD66b (clone 80H3)	Bio-Rad	MCA216T; RRID:AB_2291565
Mouse anti-human CD16 biotinylated (clone 3G8)	BD Biosciences	Cat#555405; RRID:AB_395805
AF488-conjugated mouse anti-human EpCAM (clone VU1D9)	Cell Signaling Technology	Cat#5198; RRID:AB_10692105
PE-conjugated mouse antibody anti-CD45 (clone HI30)	BD Biosciences	Cat#560975; RRID:AB_2033960
Rabbit anti-histone H3K27me3 (Western blot)	Thermo Fisher Scientific	Cat#MA5–11198; RRID:AB_2899176
Rabbit anti-histone H3K27me3 (ChIP and CUT&RUN)	Active motif	Cat#39155; RRID:AB_2561020
Rabbit anti-histone H3K9me3 (ChIP)	Abcam	Cat#ab8898; RRID:AB_306848
Rabbit anti-IgG control (clone DA1E) (CUT&RUN)	Cell Signaling Technology	Cat#66362; RRID:AB_2924329
Rabbit anti-histone H3 total	Abcam	Cat#1791; RRID:AB_302613
Rabbit anti-H3K27me3 (clone C36B11) (immunofluorescence)	Cell Signaling Technology	CST#9733; RRID:AB_2616029
APC conjugated mouse anti-human CD1d (FACS)	BioLegend	Cat#350308; RRID:AB_10642829
APC conjugated mouse anti-human CD1d (clone CD1d42)	BD Biosciences	BD#563505; RRID:AB_2738246
APC-conjugated isotype control	BD Biosciences	BD#555751
Rat inVivoMab anti-mouse CD1d (clone 20H2) (FACS for Myc-CaP cells)	Bio X Cell	#BE0179; RRID:AB_10949293
Rat InVivoPlus anti-mouse isotype control (clone HRPN) (FACS for Myc-CaP cells)	Bio X Cell	#BE0088; RRID:AB_1107775
APC conjugated goat anti-rat IgG (H+L)	Thermo Fisher Scientific	Cat#A10540
Rat anti-mouse CD16/CD32 blocking reagent (Clone: 2.4G2)	BD Biosciences	Cat#553142; RRID:AB_394657
BV510-viability dye	BD Biosciences	BD#564406; RRID:AB_2869572
APC-α-GalCer-mCD1d Tetramer	TetramerShop	Cat#MCD1d–001
BV711-conjugated anti-mouse CD69 (clone: H1.2F3)	BioLegend	Cat#104537; RRID:AB_2566120
PerCP-Cy5.5-conjugated anti-mouse TCRβ (clone: H57–597)	Biolegend	Cat#109228; RRID:AB_1575173
BV605-conjugated anti-mouse CD3e (clone: 145–2C11)	BioLegend	Cat#100351; RRID:AB_2565842
BUV395-conjugated anti-mouse NK1.1 (clone: PK136)	BioLegend	Cat#564144
BV711- conjugated anti-mouse CD8a (clone: 53–6.7)	BioLegend	Cat#100759; RRID:AB_2563510
BV650- conjugated anti-mouse CD4 (clone: RM4–5)	BioLegend	Cat#100546; RRID:AB_2562098
FITC- conjugated anti-mouse CD44 (clone: IM7)	BioLegend	Cat#103006; RRID:AB_312957
PE-Cy7- conjugated anti-mouse PD-1 (clone: RMP1–30)	BioLegend	Cat#109110; RRID:AB_572017
BV421-conjugated anti-mouse TIM3 (clone: 5D12)	BioLegend	Cat#747626
APC- conjugated anti-mouse TIGIT (clone: 4D4/mTIGIT)	BioLegend	Cat#156106; RRID:AB_2750515
BV785- conjugated anti-mouse LAG3 (clone:C9B7W)	BioLegend	Cat#125219; RRID:AB_2566571
PE- conjugated anti-mouse TNFα (clone: MP6-XT22)	BioLegend	Cat#506306; RRID:AB_315427
BV650- conjugated anti-mouse CD4 (clone: RM4–5)	BioLegend	Cat#100546; RRID:AB_2562098
BV605-conjugated anti-mouse IFNγ (clone: XMG1.2)	BioLegend	Cat#505840; RRID:AB_2734493
Biological samples		
Healthy donors for blood samples	This paper	N/A
Blood samples from patients with a diagnosis of localized of metastatic prostate cancer	This paper	N/A
Localized tumor tissue cohort (core biopsies or surgical resection)	This paper	N/A
Chemicals, peptides, and recombinant proteins		
5-azacitidine	Selleck	Cat#S1782
GSK126	Selleckchem	Cat#S7061
MNase enzyme (micrococcal nuclease)	NEB	Cat# M0247S
G418	Sigma Aldrich	Cat#G8168
Blasticidin	InvivoGen	Cat#ant–bl–05
Cell Stimulation Cocktail	eBioscience	Cat#00–4970–93
Protein Transport Inhibitor Cocktail	eBioscience	Cat#00–4980
Dynabeads MyOne Streptavidin T1	Invitrogen	Cat#65–601
Critical commercial assays		
EZ DNA methylation kit	Zymo	Cat#D5001
Zero blunt PCR cloning kit	ThermoFisher	Cat#K270020
Magnetic MyOne Carboxylic Acid Beads	Invitrogen	Cat#65011
NEBNext Ultra II DNA Library Prep Kit	NEB	Cat#E7645L
CUT&RUN Assay kit	Cell Signaling Technology	Cat#86652S
RNeasy Mini kit	QIAGEN	Cat#74104
SuperScript III One-Step qRT-PCR kit	Invitrogen	Cat#11732020
NEBuilder HiFi DNA Assembly Cloning kit	NEB	Cat#E5520S
BD Fixation/Permeabilization Solution Kit	BD Biosciences	Cat#554714
HMW DNA extraction kit	QIAGEN	Cat#67563
Rapid Barcoding Kit	Nanopore	Cat#SQK–RBK004
Deposited data		
Raw and analyzed data	This paper	GEO: GSE208449
Human reference genome NCBI build 37, GRCh37 (hg19)	Genome Reference Consortium	https://www.ncbi.nlm.nih.gov/assembly/GCF_000001405.13/
Illumina Infinium Human Methylation 450 K BeadChip	National Cancer Institute’s GDC Data Portal	https://portal.gdc.cancer.gov
DNA Methylation 450 K BeadChip datasets	National Cancer Institute’s GDC Data Portal	https://portal.gdc.cancer.gov
TCGA (PRAD samples)	CBioPortal	https://www.cbioportal.org/
Methylation profiles (TCGA cohorts, 33 cancer types)	TCGA Research Network	https://portal.gdc.cancer.gov
Genome annotations (TSS, exon, intron, intragenic regions, CpG islands (CGIs), repetitive elements and UCSC gap regions) - UCSC genome table browser	Karolchik et al, 2004	https://genome.ucsc.edu/cgi-bin/hgTables
DNA methylation datasets (colon and thyroid)	Timp et al, 2014	GEO: GSE53051
DNA methylation of normal prostate tissues and primary prostate tumors	Yu et al., 2013	Obtained from authors. https://doi.org/10.1016/j.ajpath.2013.08.018
DNA methylation of metastatic prostate tumors	Zhao et al., 2020	dbGAP: phs001648
Experimental models: Cell lines		
Human prostate cancer cell line (LNCaP, clone FGC)	ATCC	CRL–1740
Human prostate cancer cell line (VCaP)	ATCC	CRL–2876
Human prostate cancer cell line (PC3)	ATCC	CRL–1435
Human prostate cancer cell line (22Rv1 )	ATCC	CRL–2505
Murine prostate cancer line (Myc-CaP)	ATCC	CRL–3255
Normal cultured prostate epithelial cells (HPrEC)	ATCC	PCS–440–010
Benign prostatic hypertrophy cells (BPH-1)	Sigma-Aldrich	SCC256
Murine Lewis lung carcinoma cells (LLC-1)	ATCC	CRL–1642
Experimental models: Organisms/strains		
Mouse: FVB mice	Jackson Laboratory	Strain#001800
Mouse: NOD.Cg-Prkdcscid Il2rgtm1Wjl/SzJ	Jackson Laboratory	Strain#005557
Mouse: C57BL/6	Jackson Laboratory	Strain#000664
Oligonucleotides		
Primers for qRT-PCR	This paper	Table S3
Primers for Bisulfite PCR	This paper	Table S3
Recombinant DNA		
pLenti-murine Cd1d1-mGFP	Origene	Cat#MR226027L4
pLenti-C-mGFP	Origene	Cat#PS100093
pLenti-*Ifi204*-Myc-DDK-Puro	Origene	Cat#MR222527L3
pLenti-C-Myc-DDK-Puro	Origene	Cat#PS100092
lentiCRISPRv2-blast	Addgene	Cat#98293; RRID:Addgene_98293
N174-MCS	Addgene	Cat#81061; RRID:Addgene_81061
pMD2.G	Addgene	Cat#12259; RRID:Addgene_12259
psPAX2	Addgene	Cat#12260; RRID:Addgene_12260
Software and algorithms		
QUMA	Kumaki et al., 2008	http://quma.cdb.riken.jp/
ImageJ		https://imagej.nih.gov/ij/
FlowJo software (v10.4)	BD Bioscience	https://www.flowjo.com/
Trim Galore (v0.4.3)	Babraham Bioinformatics	https://github.com/FelixKrueger/TrimGalore
Tophat (v2.1.1)	Trapnell et al., 2012	https://github.com/infphilo/tophat
Samtools (v1.3.1)	Li et al., 2009	http://samtools.sourceforge.net/
HTseq (v0.6.1)	Anders et al., 2015	https://htseq.readthedocs.io/en/master/
Cufflinks (v2.1.1)	Trapnell et al., 2012	https://github.com/cole-trapnell-lab/cufflinks
R (v3.1.2)	R Core Team, 2021	https://www.R-project.org/
Graph Prism 9	GraphPad	https://www.graphpad.com/
Bismark tool (v0.17.0)	Krueger and Andrews, 2011	https://github.com/FelixKrueger/Bismark
UCSC lift-over tool	Hinrichs et al., 2006	https://genome.ucsc.edu/cgi-bin/hgLiftOver
ABSOLUTE algorithm	Carter et al., 2012	http://software.broadinstitute.org/cancer/cga/absolute_download
Molecular Signatures Database (MSigDB) (v7.2)	Broad Institute & UC San Diego Subramanian, Tamayo et al., 2005 Liberzon et al., 2011	https://www.gsea-msigdb.org/gsea/msigdb/index.jsp
Bioconductor package regioneR (v1.18.1) with overlapPermTest function	Gel et al., 2016	https://www.bioconductor.org/packages/release/bioc/html/regioneR.html
Ginkgo	Garvin et al., 2015	http://qb.cshl.edu/ginkgo
InferCNV (V 1.10.1)	Tickle et al., 2019	https://github.com/broadinstitute/infercnv
BWA men	Li and Durbin, 2009	https://github.com/lh3/bwa
Sambamba	Tarasov et al., 2015	https://github.com/biod/sambamba
MACS2 (v2.0.10)	Zhang et al., 2008	https://github.com/macs3-project/MACS
DeepTools	Ramirez et al., 2016	https://github.com/deeptools/deepTools
phyper R function	Johnson et al., 1992	https://www.R-project.org/
ONT Albacore software (v2.3.1)	Oxford Nanopore Technologies	https://nanoporetech.com/community
Nanopolish software (v0.10.2)	Simpson et al., 2017	https://github.com/nanoporetech/nanopolish
PRROC R-package	Grau et al., 2015	https://cran.r-project.org/web/packages/PRROC/index.html
BioRender	BioRender	https://www.biorender.com/
Other		
Lipofectamine 2000 reagent	Invitrogen	Cat#1668019
LentiX concentrator	Clontech Labs	NC0448638
Polybrene	Santa Cruz	sc–134220
Nanopore MinION device with R9.4 flowcell	Oxford Nanopore Technologies	FLO–MIN106D
HRP conjugated secondary antibodies	Bio-rad	Cat#5196–2504
Laemmli buffer	Sigma	S3401–10VL

## Highlights

1. 40 core hypomethylated domains across prostate CTCs arise early in tumorigenesis.
2. Hypomethylation silences immune-related genes, sparing adjacent proliferation genes.
3. The *CD1A-IFI16* immune locus is consistently silenced by hypomethylation in cancer.
4. Hypomethylated domains detected in CTC-enriched blood in localized prostate cancer.
