# Supplementary material for: DNA hypomethylation silences antitumor immune genes in early prostate cancer and CTCs
Source: Cell. Author manuscript; Available in PMC 2023 Aug 18. (PMC10436379; doi:10.1016/j.cell.2023.05.028)

Figure S1. Single-cell transcriptome and DNA copy number analysis of prostate CTCs, related to Figure 1.

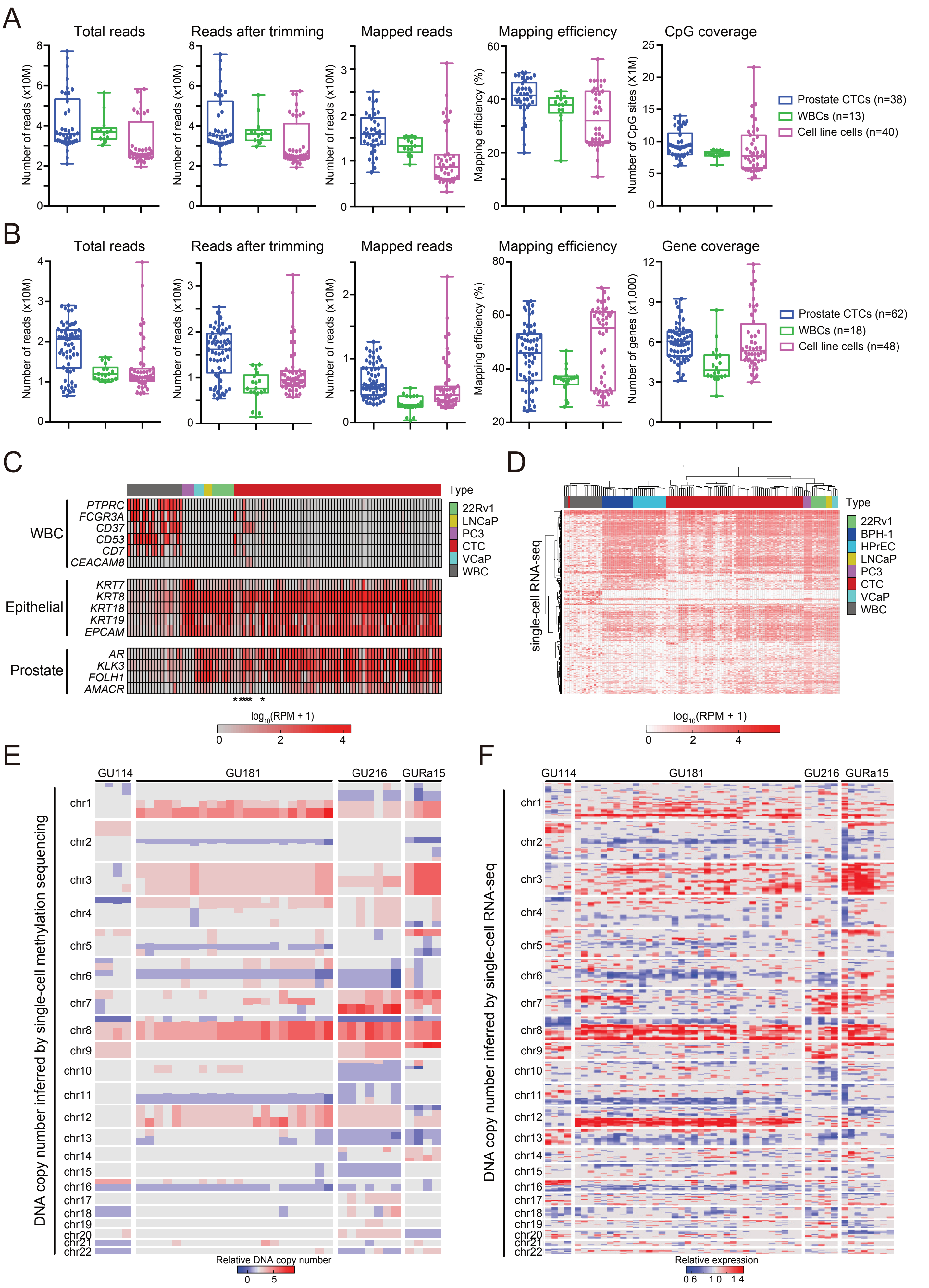

Supplement: 6 — Figure S1. Single-cell transcriptome and DNA copy number analysis of prostate CTCs, related to Figure 1. (A-B) Boxplots showing sequencing quality parameters of single-cell DNA methylation samples (panel A) and single-cell RNA-seq samples (panel B). (C) Heatmap showing marker gene expression of the single cells sequenced in this study. CTCs have high expression of epithelial and prostate lineage markers, and absent expression of leukocyte (WBC) markers. Single WBCs that persisted after processing through the microfluidic device are shown as negative controls, and single cells from prostate cancer cell lines are used as positive controls. Asterisks denote a small number of CTCs with potential contamination by WBCs, which were excluded from analysis. (D) Heatmap showing unsupervised hierarchical clustering of single-cell RNA-seq. Three major clusters are defined (upper dendrogram): WBC; normal prostate cell line cells (HPrEC and BPH-1); and prostate CTCs together with four prostate cancer cell lines (PC3, LNCaP, VCaP and 22Rv1). (E-F) Heatmaps showing matched DNA copy number of CTCs inferred from single-cell DNA methylation sequencing data (panel E) and from single-cell RNA-seq data (panel F). [file NIHMS1910396-supplement-6.pdf]
