# Supplementary figures and images for: DNA hypomethylation silences antitumor immune genes in early prostate cancer and CTCs
Source: Cell. Author manuscript; Available in PMC 2023 Aug 18. (PMC10436379; doi:10.1016/j.cell.2023.05.028)

Figure S2. DNA methylation analysis of single prostate CTCs, related to Figure 1.

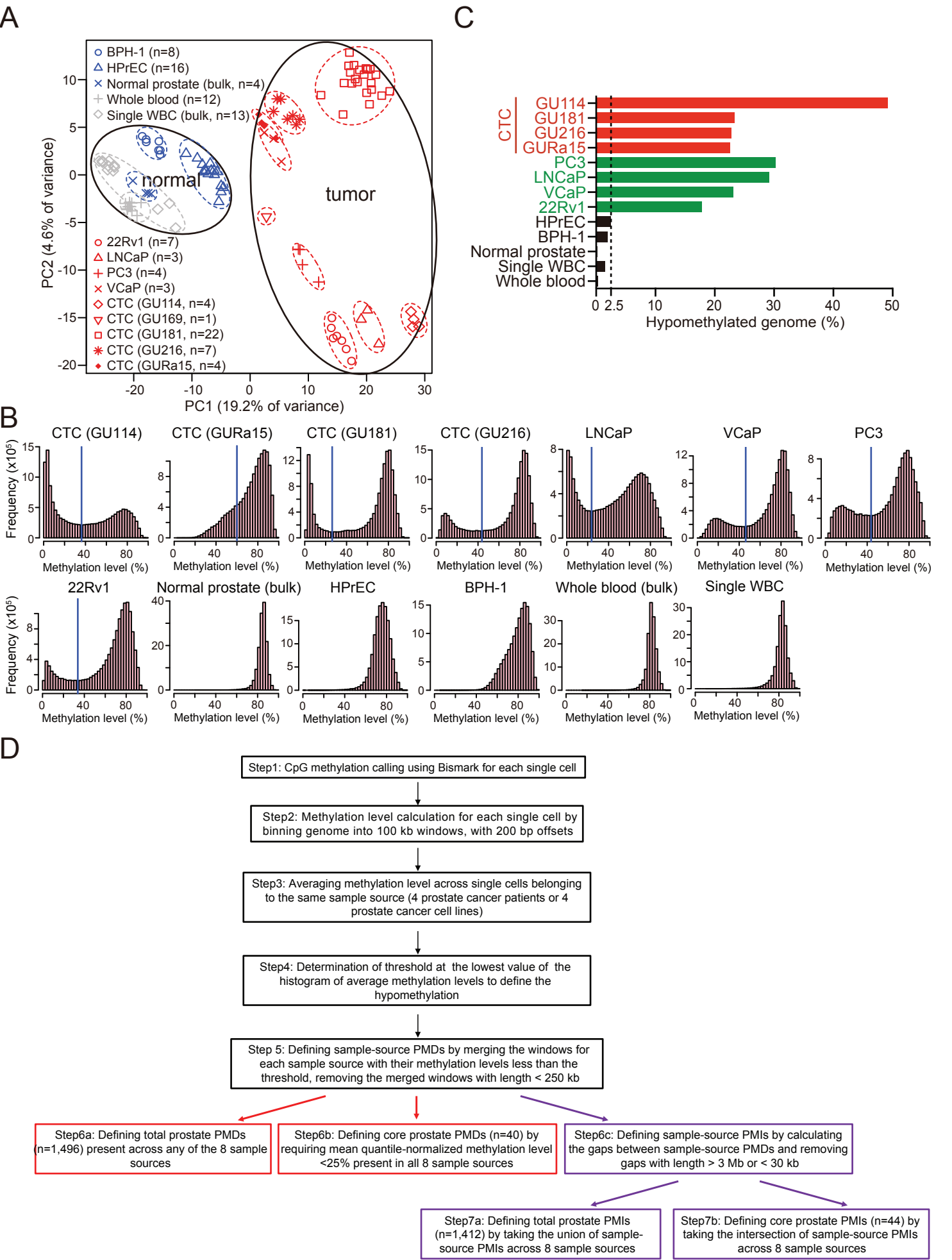

Supplement: 7 — Figure S2. DNA methylation analysis of single prostate CTCs, related to Figure 1. (A) PCA analysis of promoter methylation in single prostate CTCs, and single cells from prostate cancer cell lines (22Rv1, LNCaP, PC3 and VCaP), non-transformed prostate epithelial cell lines (HPrEC and BPH-1), residual WBCs following microfluidic processing, and normal prostate tissues. All prostate tumor cells cluster separately from both non-transformed prostate cells and from normal leukocytes. (B) Histograms showing distribution of methylation level within each 100 kb window, with coverage of at least 10 CpGs at 200bp offsets across the genome, representing averages from single-cell data for patient-derived CTCs (grouped by patient: GU114, GU181, GU216, GURa15), prostate cancer cell lines (LNCaP, VCaP, PC3 and 22Rv1), normal prostate tissue, non-transformed prostate epithelial cell lines (HPrEC and BPH-1), whole blood (representing all hematopoietic lineages) and leukocytes (WBC). The blue vertical line in each cancer-related specimen is the threshold set to score hypomethylation in that cell type (i.e., every 100 kb window with methylation levels below that threshold is defined as hypomethylated). (C) Bar graph showing the fraction of the genome that is hypomethylated in patient-derived CTCs, prostate cancer cell lines, and normal cell lines or tissues. All tumor samples have 20–40% of their genome classified as hypomethylated, while the normal samples have <2.5%. N.P., normal prostate. (D) Flowchart depicting the key steps of definition of prostate PMDs and PMIs (see Methods). [file NIHMS1910396-supplement-7.pdf]

Figure S3. Chromatin silencing marks and size of core PMDs and core PMIs, related to Figure 2.

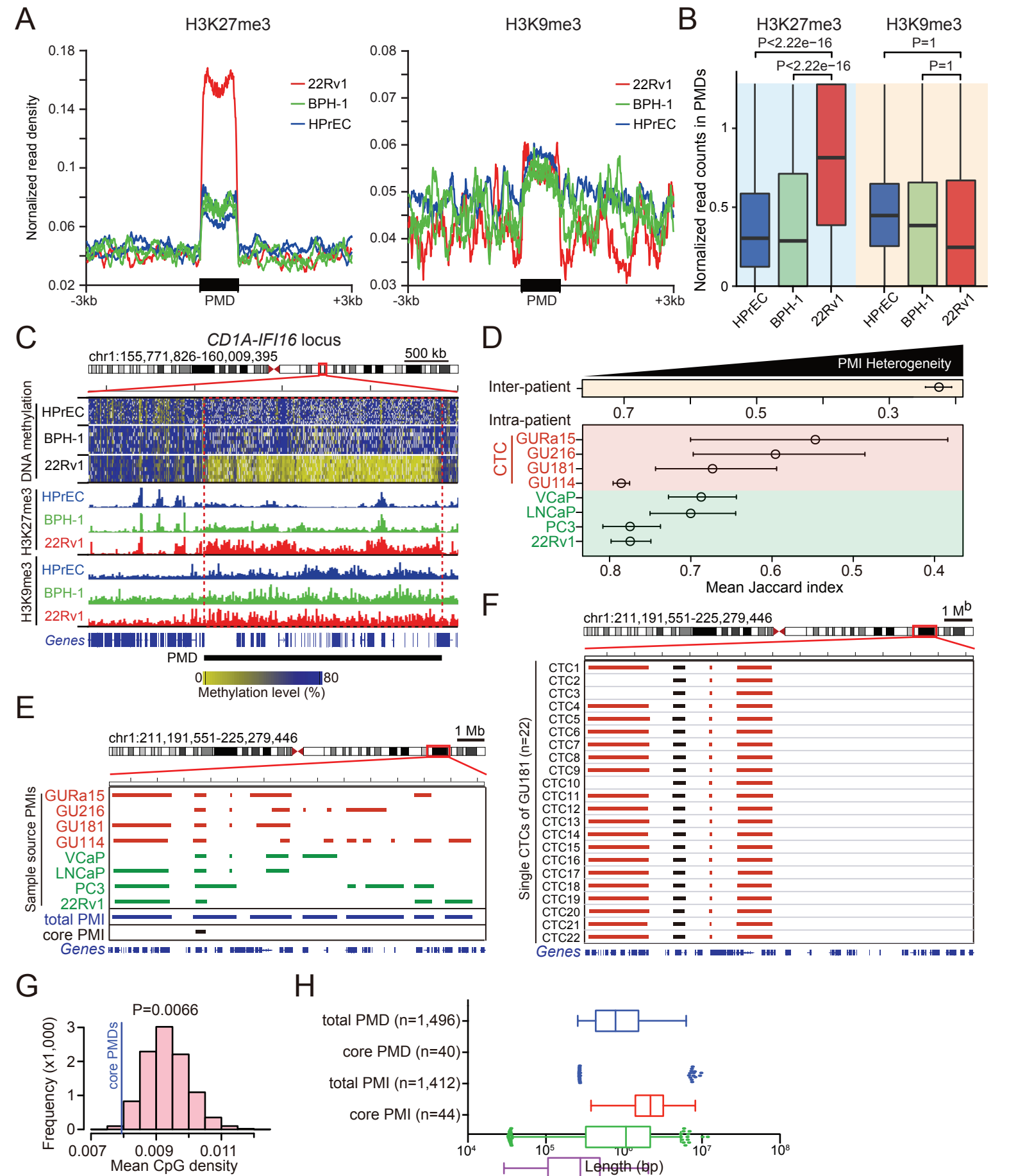

Supplement: 8 — Figure S3. Chromatin silencing marks and size of core PMDs and core PMIs, related to Figure 2. (A) Line plots showing differential enrichment for H3K27me3 marks at PMDs in prostate cancer cells (22Rv1, red) compared with non-transformed prostate epithelial cell lines (BPH-1, green and HPrEC, blue) (left panel). In contrast, there is no significant difference in the abundance of H3K9me3 at PMDs between cancer cells and normal cells (right panel). (B) Boxplot quantifying the enrichment for H3K27me3 at all prostate PMDs in 22Rv1 prostate cancer cells, compared with normal prostate HPrEC and BPH-1 cells. No enrichment is observed for H3K9me3. P-value assessed by one-tailed Student’s t test. (C) IGV screenshot (hg19) of the CD1A-IFI16 locus at chromosome 1, showing DNA hypomethylation (shade yellow) in 22Rv1 prostate cancer cells (red), compared with HPrEC and BPH-1 prostate epithelial cells (blue and green). The CD1A-IFI16 locus also shows enrichment for H3K27me3 chromatin silencing marks in prostate cancer cells, compared with normal prostate cells, but no such differential abundance for H3K9me3 silencing marks. (D) Inter- and intra-patient heterogeneity analysis of PMIs among prostate CTCs and single cells from prostate cancer cell lines. Mean Jaccard index is used to indicate the heterogeneity, with higher mean Jaccard index score indicating less heterogeneity among samples assayed. Error bar indicates mean with 95% CI. (E-F) IGV representation (hg19) of total PMIs and core PMIs at a chromosome 1 locus, across 8 sample sources (4 prostate patients and 4 prostate cancer cell lines). Total PMIs (blue) are the union of all PMIs defined in each sample source, while core PMIs (black) are those shared across all 8 sample sources (panel E); representation of PMIs from the single-cell components of an individual sample source (22 CTCs from patient GU181) showing a core PMI (black) shared across all sample sources and neighboring non-core PMIs (red) that are shared by >85% of [file NIHMS1910396-supplement-8.pdf]
