# Supplementary material for: DNA hypomethylation silences antitumor immune genes in early prostate cancer and CTCs
Source: Cell. Author manuscript; Available in PMC 2023 Aug 18. (PMC10436379; doi:10.1016/j.cell.2023.05.028)

Figure S4. Demethylation of *CD1A-IFI16* locus at early stage of prostate tumorigenesis, related to Figure 4.

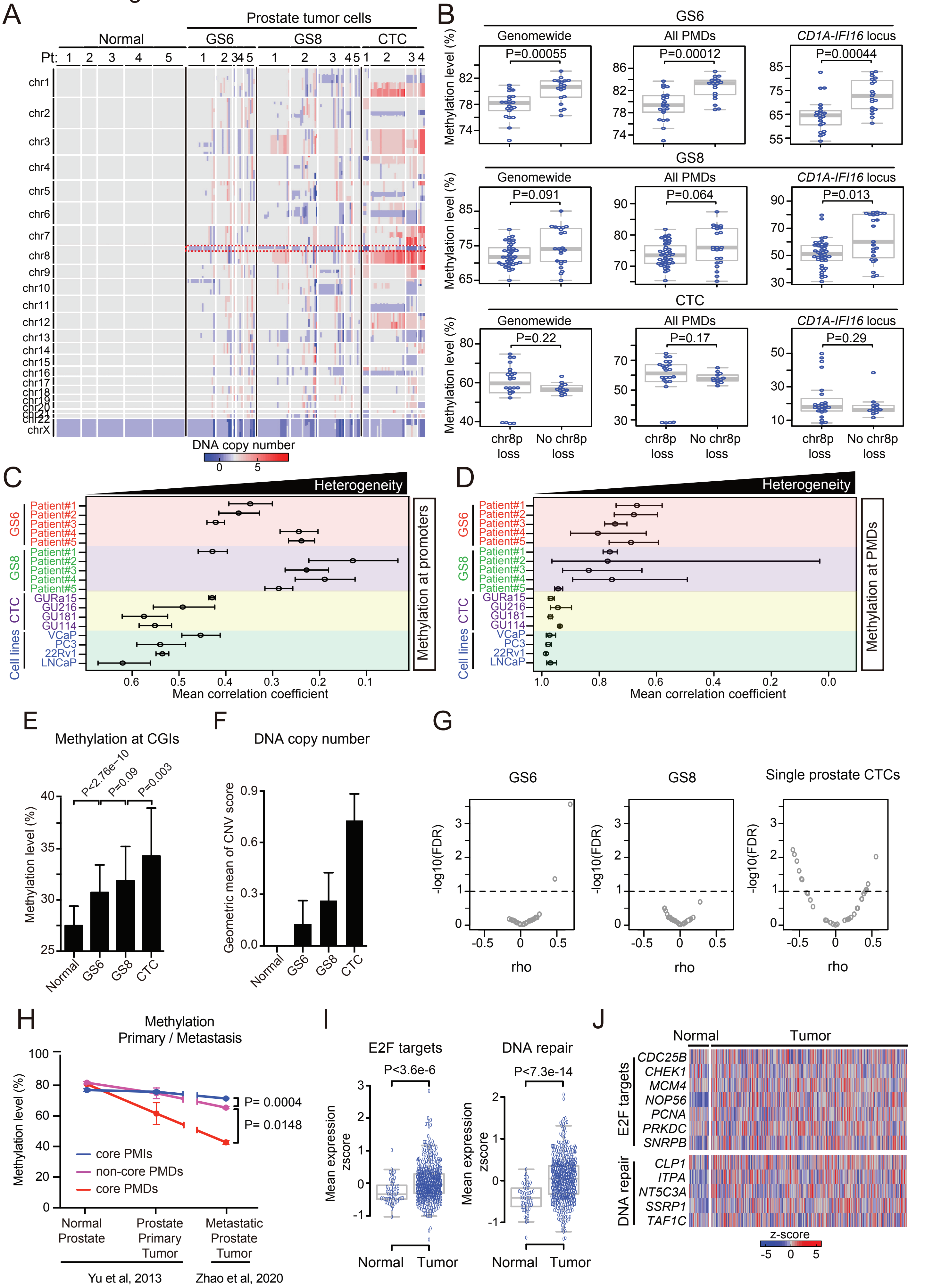

Supplement: 9 — Figure S4. Demethylation of CD1A-IFI16 locus at early stage of prostate tumorigenesis, related to Figure 4. (A) Heatmap showing DNA copy number variation (CNVs) within single cells retrieved from adjacent normal tissues, low-grade localized prostate cancer (GS6), high-grade localized prostate cancer (GS8), and metastatic prostate cancer (CTCs). Single-cell DNA methylation sequencing data were used to infer CNVs. Box marked by dashed red line denotes chromosomal deletion of the chr8p locus, which appears as the earliest and most consistently observed CNV in early prostate cancer. (B) Boxplots showing concordance of hypomethylation and chr8p deletion within single cancer cells at the one of the earliest stage of prostate tumorigenesis (GS6), across the genome, at all PMDs, and at the CD1A-IFI16 locus. The correlation is lost at more advanced stages of prostate cancer (GS8 and CTCs), when CNV and hypomethylation are pronounced and distributed across the genome. P-value, all assessed by Wilcoxon test. (C-D) Heterogeneity of promoter methylation (panel C) and PMD methylation (panel D) within individual cells from localized prostate cancer (GS6 and GS8), metastatic prostate cancer (CTCs) and prostate cancer cell lines, measured by mean correlation coefficient, and showing the relative uniformity of PMD hypomethylation in prostate cancer, compared with promoter hypermethylation. Error bar denotes mean with 95% CI. (E) Bar plots showing gradual increase of methylation at CpG islands (CGIs) during prostate cancer progression. Error bar denotes mean with SD. P-value was assessed by two-tailed Student’s t test. (F) Quantitation of DNA copy number alterations during prostate tumorigenesis, with normal prostate showing no CNV, and gradual increase in CNV from GS6, to GS8, and CTCs. Error bar indicates geometric mean with 95% CI. (G) Scatter plot showing correlation between DNA copy number alterations and DNA methylation changes in GS6 and GS8 tumors and in prostate CTCs. Each do [file NIHMS1910396-supplement-9.pdf]
