# Supplementary material for: DNA hypomethylation silences antitumor immune genes in early prostate cancer and CTCs
Source: Cell. Author manuscript; Available in PMC 2023 Aug 18. (PMC10436379; doi:10.1016/j.cell.2023.05.028)

Figure S5. DNA methylation analysis at the *CD1A-IFI16* locus in prostate and other cancers, related to Figure 4.

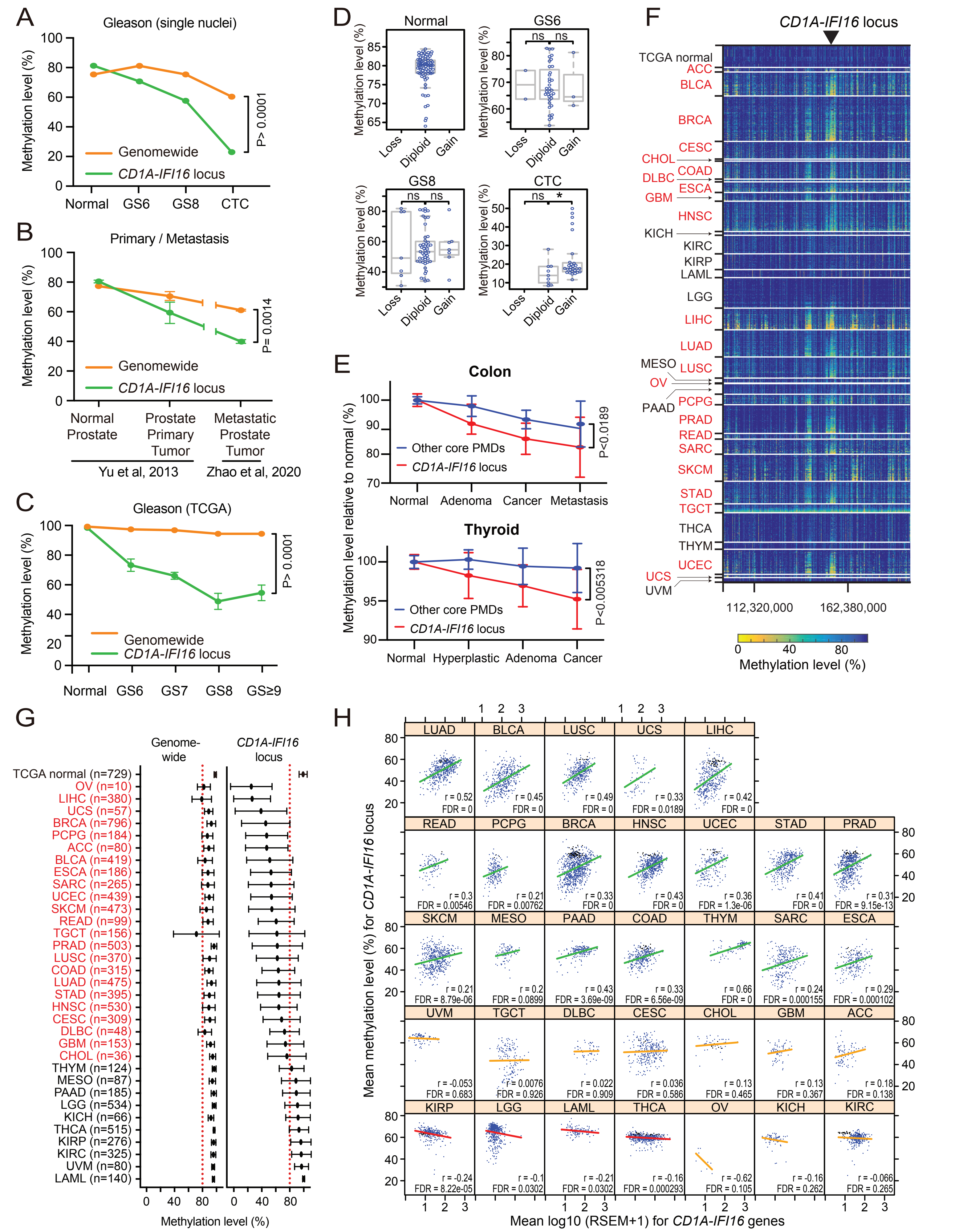

Supplement: 10 — Figure S5. DNA methylation analysis at the CD1A-IFI16 locus in prostate and other cancers, related to Figure 4. (A-C) DNA methylation changes as a function of Gleason score, in microdissected single nuclei from surgically resected specimens of localized prostate cancer (A), in samples derived from two independent public datasets of prostate tumors (normal prostate tissue and primary prostate tumors derived from Yu et al, 2013, and metastatic prostate tumors derived from Zhao et al, 2020) (B) and from Gleason-annotated TCGA prostate tumor specimens (C). Line plots show a marked loss of DNA methylation at the CD1A-IFI16 locus (green line), in contrast to genomewide DNA methylation (orange line). Of note, (A) and (B) data derived from WGBS, and (C) data from Infinium Human Methylation 450K BeadChip. Error bar denotes mean with SEM. Statistical analysis of DNA methylation curves using longitudinal linear mixed effects model, by which tumor progression (or Gleason Score) x methylation domains was tested. (D) Boxplots showing absence of significant methylation changes across single cells (normal, GS 6, GS 8, CTCs) representing different grades of prostate cancer, as a function of DNA copy number variation at the CD1A-IFI16 locus. ns, not significant; *P<0.05, assessed by Wilcoxon test. (E) Line graphs showing earlier demethylation at the CD1A-IFI16 locus, compared with other core PMDs, during colon cancer and thyroid cancer progression. Error bar denotes mean with SD. P-value, assessed by two-tailed Student’s t test. (F) IGV screenshot (hg19) showing DNA methylation at a region of chromosome 1 encompassing the CD1A-IFI16 locus, across 33 different cancer types (TCGA). 23 (denoted in red) show hypomethylation (<80%) of the CD1A-IFI16 locus. Abbreviations for cancer types: ACC, Adrenocortical Carcinoma; BLCA, Bladder Urothelial Carcinoma; BRCA, Breast Invasive Carcinoma; CESC, Cervical Squamous Cell Carcinoma and Endocervical Adenocarcinoma; CHOL, Cholangiocarcinoma; COAD, [file NIHMS1910396-supplement-10.pdf]
