# Supplementary material for: DNA hypomethylation silences antitumor immune genes in early prostate cancer and CTCs
Source: Cell. Author manuscript; Available in PMC 2023 Aug 18. (PMC10436379; doi:10.1016/j.cell.2023.05.028)

Figure S6. Chromatin silencing marks and transcriptional changes at the *CD1A-IFI16* locus, related to Figure 4.

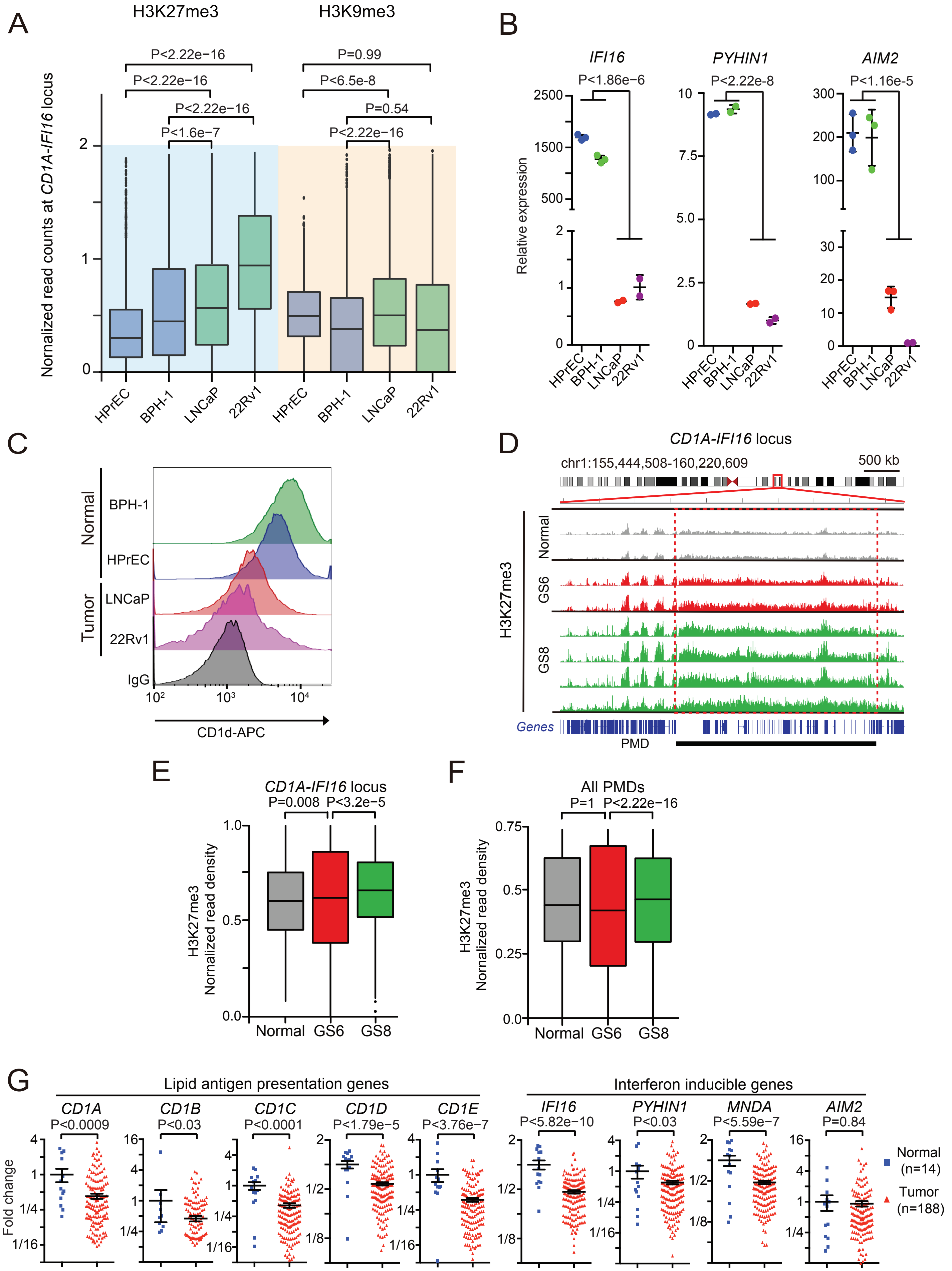

Supplement: 11 — Figure S6. Chromatin silencing marks and transcriptional changes at the CD1A-IFI16 locus, related to Figure 4. (A) Boxplots quantifying increased H3K27me3 marks, but unchanged H3K9me3, in prostate cancer cells compared with normal cells, at the CD1A-IFI16 locus, using Cut and Run assays. Two prostate cancer cell lines (LNCaP and 22Rv1) are compared with the two non-transformed prostate epithelial cell lines (HPrEC and BPH-1). P-value is assessed by one-tailed Student’s t test. (B) Plots showing reduced expression (qRT-PCR) of interferon inducible genes at the CD1A-IFI16 locus (IFI16, PYHIN1 and AIM2) in two prostate cancer cell lines (LNCaP and 22Rv1), compared with two non-transformed epithelial prostate cell lines (HPrEC and BPH-1). Error bar denotes mean with SD. P-value assessed by two tailed Student’s t test. (C) Flow cytometric quantitative analysis of CD1d expression in two prostate epithelial cell lines (BPH-1 and HPrEC), compared with two prostate cancer cell lines (LNCaP and 22Rv1), showing reduced CD1d expression in the tumor cells. Cells were incubated with the APC conjugated anti-human CD1d or with the APC-conjugated isotype control IgG (shown in grey). (D) IGV screenshot (hg19) showing enrichment for H3K27me3 ChIP-seq signal at the CD1A-IFI16 locus, as early as GS 6 during early prostate tumorigenesis. H3K27me3 ChIP-seq is shown for two normal prostate tissues (grey tracks), two GS 6 tumor tissues (red tracks) and four GS 8 tumor tissues (green tracks). (E-F) Boxplots showing quantitative enrichment for the chromatin silencing mark H3K27me3 during progression from normal prostate epithelium to stages of localized prostate cancer (GS 6, GS 8). Increased H3K27me3 is evident as early as GS 6 (panel E; representative IGV track (hg19) shown in panel D), whereas all PMDs do not show statistically significant increased deposition of H3K27me3 until GS 8 (panel F). P-value all assessed by one-tailed Student’s t test. (G) Plots showing reduced expression of all [file NIHMS1910396-supplement-11.pdf]
