# Supplementary material for: DNA hypomethylation silences antitumor immune genes in early prostate cancer and CTCs
Source: Cell. Author manuscript; Available in PMC 2023 Aug 18. (PMC10436379; doi:10.1016/j.cell.2023.05.028)

Figure S7. Functional recapitulation of hypomethylation-associated silencing at *CD1A-IFI16* locus, related to Figure 4.

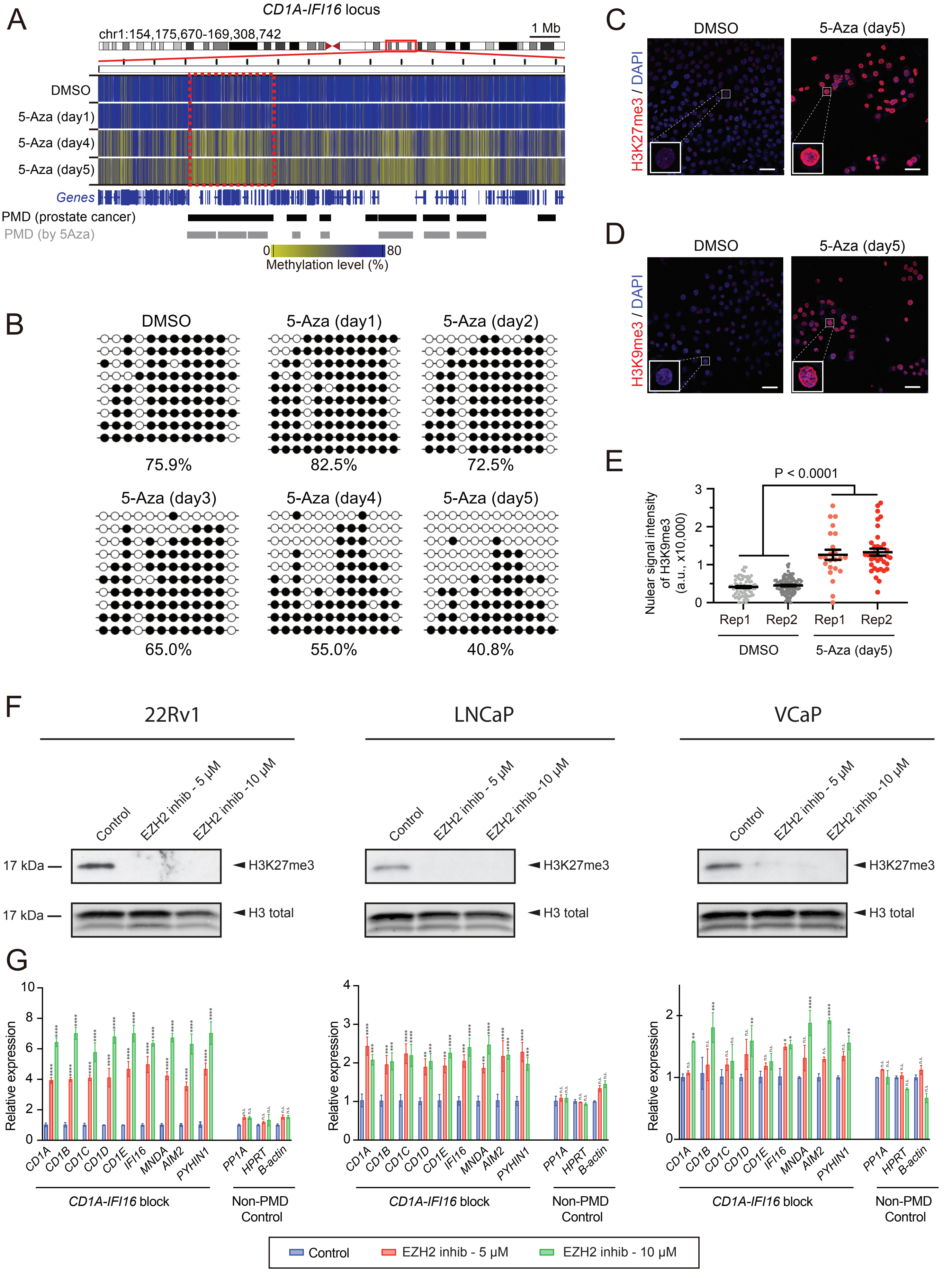

Supplement: 12 — Figure S7. Functional recapitulation of hypomethylation-associated silencing at CD1A-IFI16 locus, related to Figure 4. (A) IGV screenshot (hg19) of bulk DNA methylation profile at the CD1A-IFI16 locus (delineated by hashed red box) showing DNA demethylation in the human prostate epithelial cells BPH-1 following 4–5 days of treatment with 5-azacytidine, compared with DMSO controls and day 1 after treatment. (B) Lollipop graph showing region within the CD1A-IFI16 locus (12 CpG sites over 395 bp), using bisulfite PCR coupled with Sanger sequencing from BPH-1 cells at serial timepoints after treatment with 5-azacytadine. Methylated CpGs (black circles), unmethylated CpGs (open circles), with mean DNA methylation fraction indicated below each panel. € Representative confocal microscopic images of BPH-1 cells showing increased nuclear abundance of the H3K27me3 chromatin silencing mark, following 5 days of treatment with 5-azacytidine (versus DMSO control). DNA content is labelled with DAPI (blue). Red fluorescence indicated H3K27me3. One magnified representative nucleus is shown in the lower left corner. Bar 50 μM. (D) Representative confocal microscopic images of BPH-1 cells showing increased nuclear abundance of the H3K9me3 chromatin silencing mark, following 5 days of treatment with 5-azacytidine (versus DMSO control). DNA content is labelled with DAPI (blue). Red fluorescence indicated H3K9me3. One magnified representative nucleus is shown in the lower left corner. Bar 50 μ€(E) Quantitation of confocal microscopic imaging of mean H3K9me3-related fluorescence intensity within single-cell nuclei (quantitation using ImageJ software, see methods). Error bar denotes mean with SEM. P-value assessed by two tailed Student’s t test. (F-G) Induction of genes residing at the CD1A-IFI16 locus in three human prostate cancer cell lines (22Rv1, LNCaP and VCaP), which harbor PMD hypomethylation and H3K27me3 deposition, following their treatment of with the EZH2 inhibitor GSK126 for s [file NIHMS1910396-supplement-12.pdf]
