# Supplementary material for: DNA hypomethylation silences antitumor immune genes in early prostate cancer and CTCs
Source: Cell. Author manuscript; Available in PMC 2023 Aug 18. (PMC10436379; doi:10.1016/j.cell.2023.05.028)

Figure S8. Re-expression of the lipid antigen presentation gene *Cd1d1* or the interferon inducible gene *Ifi204* suppresses tumorigenesis in immune competent murine prostate cancer models, related to Figure 5.

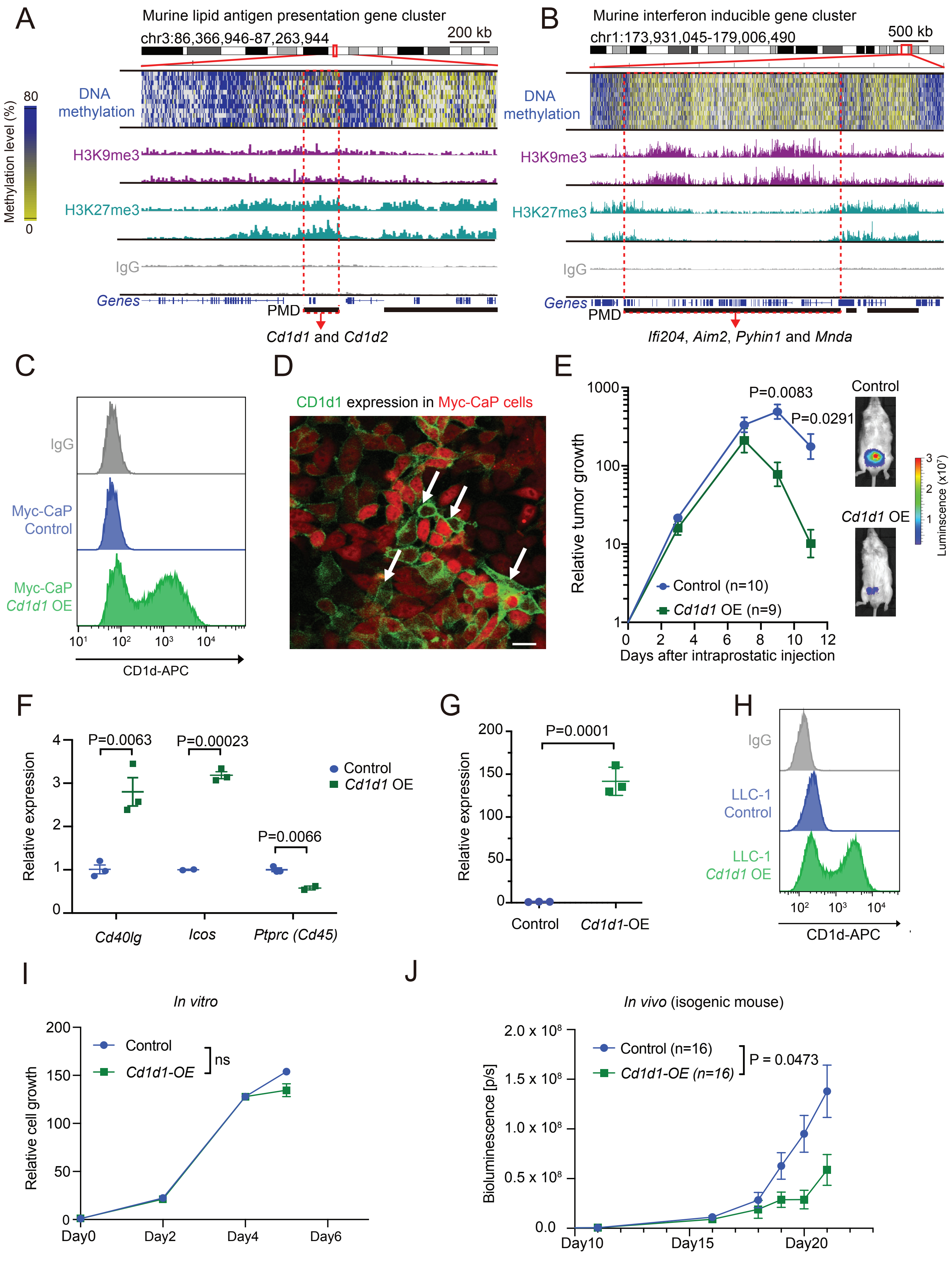

Supplement: 13 — Figure S8. Re-expression of the lipid antigen presentation gene Cd1d1 or the interferon inducible gene Ifi204 suppresses tumorigenesis in immune competent murine prostate cancer models, related to Figure 5. (A-B) IGV screenshots (mm9) showing DNA hypomethylation (shaded yellow) and occupancy of the repressive histone marks H3K27me3 and H3K9me3 in mouse Myc-CaP prostate cancer cells, at the two murine loci that are orthologous to human CD1A-IFI16: Cd1d1 and Cd1d2 genes are clustered on mouse chromosome 3 (panel A), and interferon-inducible genes Ifi204, Aim 2, Pyhin1 and Mnda are clustered on mouse chromosome 1 (panel B). H3K9me3, H3K27me3 and IgG (control) are shown with two biological replicates. DNA methylation derived from single-cell whole genome bisulfite sequencing of 12 single Myc-CaP cells. (C) Flow cytometric analysis of lentivirally-mediated ectopic Cd1d1 overexpression (OE) in Myc-CaP cells, showing cell surface localization of the encoded protein, compared with empty vector transfected control and IgG staining control. (D) Confocal microscopic image showing cell surface localization of CD1d after ectopic expression in Myc-CaP cells. Green florescence indicates CD1d expression (white arrows), Myc-CaP cells are labeled with mCherry. Bar, 20 μM. (E) In vivo intraprostatic orthotopic tumorigenesis assay, showing the relatively rapid clearance of Myc-CaP cancer cells with ectopic Cd1d1 expression, compared with parental control. Representative mouse images at day 9 are shown. Error bar denotes mean with SEM. P-value, assessed by two-tailed Student’s t test. (F) Plot showing quantitative (qRT-PCR) gene expression difference of NKT cell-specific RNA markers (Cd40lg and Icos) and pan-leucocyte marker (Ptprc, CD45), between tumors generated by Myc-CaP cells with restored Cd1d1 expression (0E) versus mock-transfected controls. P-value is assessed by two-tailed Student’s t test. (G) qRT-PCR quantitation showing expression of Cd1d1 expression in LLC-1 cells after le [file NIHMS1910396-supplement-13.pdf]
