# Supplementary material for: DNA hypomethylation silences antitumor immune genes in early prostate cancer and CTCs
Source: Cell. Author manuscript; Available in PMC 2023 Aug 18. (PMC10436379; doi:10.1016/j.cell.2023.05.028)

Figure S9. Flow cytometric analysis of immune infiltration in subcutaneous Myc-CaP-derived tumors, related to Figure 5.

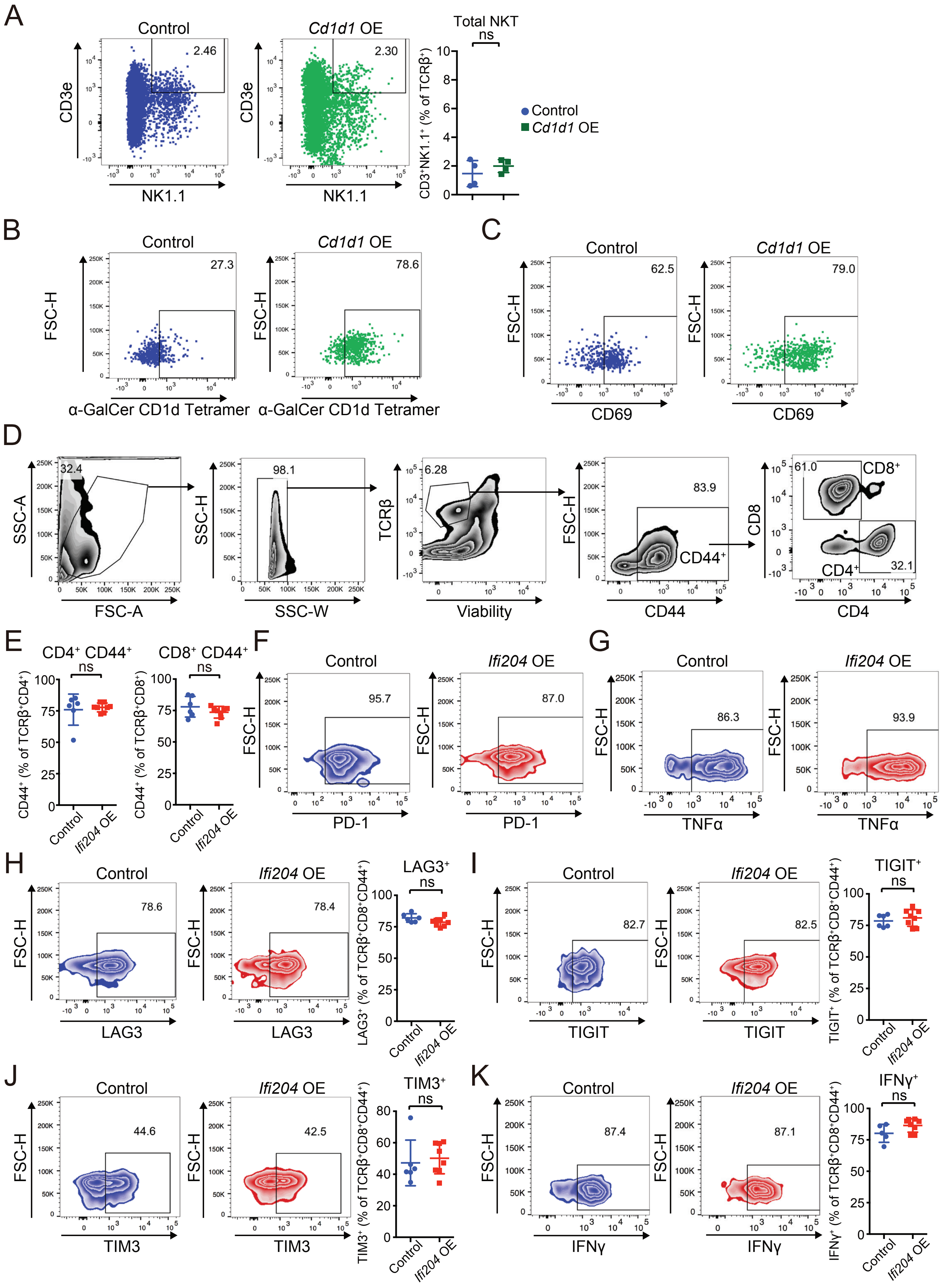

Supplement: 14 — Figure S9. Flow cytometric analysis of immune infiltration in subcutaneous Myc-CaP-derived tumors, related to Figure 5. (A) Flow cytometric analysis showing no significant difference in tumor infiltration by CD3e+NK1.1+ double positive cells (total NKT cells, not CD1d-restricted) in tumors derived in immune competent isogenic mice by Myc-CaP cells with Cd1d1 overexpression (OE) or mock-transduced controls. Quantitative analysis in right panel. ns, not significant; P-value is assessed by two-tailed Student’s t test. (B-C) Flow cytometric analysis showing enrichment of CD1d-restricted NKT cells (marked by α-GalCer CD1d Tetramer; panel B) and activated NKT cells (marked by CD69 expression; panel C) in tumors derived from Myc-CaP cells with Cd1d1 overexpression (OE), compared to mock-transduced controls. Quantitation of positive cells (percent) shown in upper right. (D) Gating strategy for scoring of different cell surface markers of infiltrated T cells in one representative tumor sample. (E) No difference in percent of CD44 expressing CD4+ or CD8+ T cells in parental control versus Ifi204 expressing tumors. ns, not significant, assessed by two tailed Student’s t test. (F-G) FACS plots showing downregulation of PD-1 cell surface expression (panel F) and upregulation of TNFα (panel G) in CD8+ T cells recovered from Myc-CaP-derived tumor with overexpression (OE) of Ifi204, compared with mock-transduced controls. (H-K) No difference in LAG3 (panel H), TIGIT (panel I), TIM3 (panel J) and IFNγ (panel K) expression in CD8+ T cells in control versus Ifi204 expressing tumors. Error bar denotes mean with SD. ns, not significant, assessed by two tailed Student’s t test. [file NIHMS1910396-supplement-14.pdf]
